# Light-fueled dynamic covalent crosslinking of single polymer chains in non-equilibrium states†

**DOI:** 10.1039/d0sc05818a

**Published:** 2020-11-17

**Authors:** Daniel Kodura, Hannes A. Houck, Fabian R. Bloesser, Anja S. Goldmann, Filip E. Du Prez, Hendrik Frisch, Christopher Barner-Kowollik

**Affiliations:** School of Chemistry and Physics, Queensland University of Technology (QUT) 2 George Street Brisbane QLD 4000 Australia a.goldmann@qut.edu.au h.frisch@qut.edu.au christopher.barnerkowollik@qut.edu.au; Centre for Materials Science, Queensland University of Technology (QUT) 2 George Street Brisbane QLD 4000 Australia; Polymer Chemistry Research Group, Centre of Macromolecular Chemistry (CMaC), Department of Organic and Macromolecular Chemistry, Ghent University Krijgslaan 281 S4-bis 9000 Gent Belgium Filip.DuPrez@UGent.be

## Abstract

While polymer synthesis proceeds predominantly towards the thermodynamic minimum, living systems operate on the reverse principle – consuming fuel to maintain a non-equilibrium state. Herein, we report the controlled formation of 3D macromolecular architectures based on light-fueled covalent non-equilibrium chemistry. In the presence of green light (525 nm) and a bivalent triazolinedione (TAD) crosslinker, naphthalene-containing polymers can be folded into single chain nanoparticles (SCNPs). At ambient temperature, the cycloaddition product of TAD with naphthalene reverts and the SCNP unfolds into its linear parent polymer. The reported SCNP is the first example of a reversible light triggered folding of single polymer chains and can readily be repeated for several cycles. The folded state of the SCNP can either be preserved through a constant supply of light fuel, kinetic trapping or through a chemical modification that makes the folded state thermodynamically favored. Whereas small molecule bivalent TAD/naphthalene cycloaddition products largely degraded after 3 days in solution, even in the presence of fuel, the SCNP entities were found to remain intact, thereby indicating the light-fueled stabilization of the SCNP to be an inherent feature of the confined macromolecular environment.

## Abstract 2

Synthetic polymers consume green light as fuel for intramolecular crosslinking, yielding non-equilibrium single chain nanoparticles that can be light-stabilised, kinetically and chemically trapped, or else unfold in the absence of light fuel.

## Graphical abstract

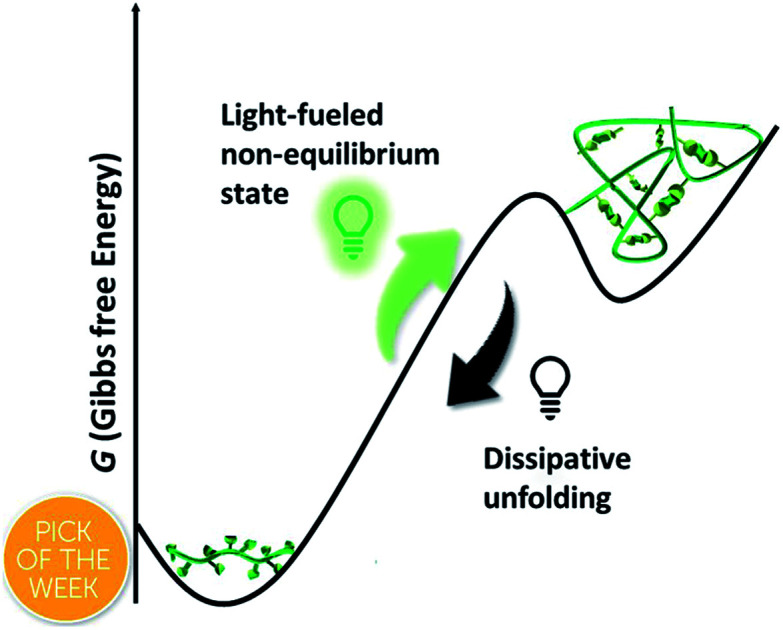

## Introduction

**Scheme 1:**
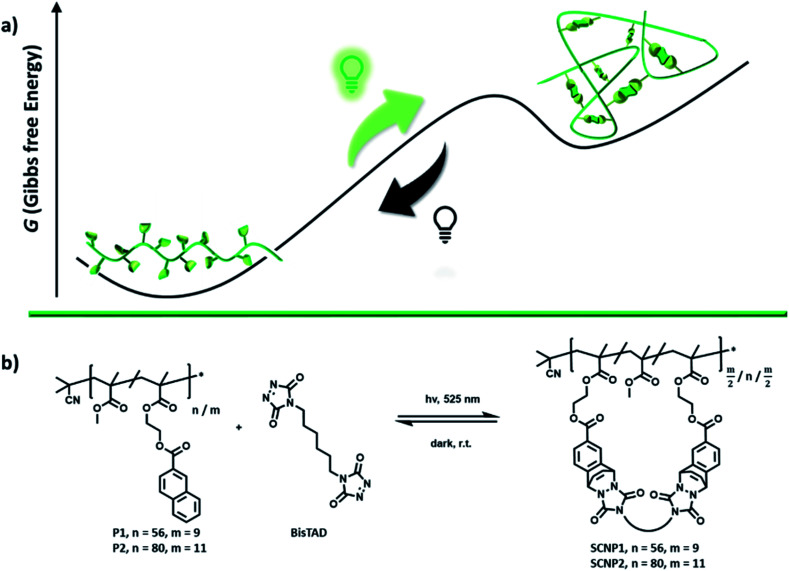
(a) Schematic illustration of the reversible folding process of a linear polymer into a single-chain nanoparticle, using green light as a fuel. As long as the light source is switched on, fuel is consumed and the architecture is in a folded non-equilibrium state. If the light source is turned off, the architecture unfolds again and reaches the equilibrium point. (b) Chemical structures of the statistical RAFT-copolymers P1 and P2 of functional naphthalene monomers and methyl methacrylate, used in the folding/unfolding process with BisTAD crosslinkers.

During the last century, the majority of chemical transformations was covalently driven, aiming to approach a thermodynamic minimum.^1^ Chemistry at equilibrium has eminent advantages, such as facile synthesis of materials, straightforward analysis and long lasting stability, yet limits the ability of a material to reform – leading to challenging repair and expensive recycling.^2^ In contrast to equilibrium driven chemistry, all living organisms consume fuel to operate far from equilibrium states – otherwise life could not exist.^3,4^ The vast potential of dynamic non-equilibrium materials has inspired a wide range of synthetic systems over the last decade comprising life-like properties.^5^ To design chemical systems that consume fuel to acquire a higher energetic state and dissipate back into the original state in the absence of fuel, the use of natural building blocks provides an attractive avenue.^6^ The teams of Walther,^7,8^ Hermans^9^ or Bhatia^10^ for instance, developed such life-like systems based on reactions of competing enzymes. However, the design of artificial systems is not constrained to the exclusive use of natural building blocks, yet can access the entire tool box of synthetic chemistry.^11^ Thus far, methylations,^12,13^ thiol–disulfide exchange,^14^ oxidation of aldehydes into α-hydroxy sulfonates,^15^ thiol–enone chemistry,^16^ alkene metathesis,^17^ metal coordination,^18^ imine bond chemistry,^19^ thiol–ester exchange^20^ and esterifications^21,22^ have been explored in synthetic reaction cycles, which consume fuel to generate a chemical product that in its absence dissipates back into its ground state. In such examples, the resulting non-equilibrium molecules have altered propensities for supramolecular interactions, affording higher order supramolecular architectures that are exclusively formed in the presence of fuel. For the same purpose, the fabrication of covalently connected macromolecular architectures, however, is far less explored.^7^ Especially in the absence of catalysts, dynamic covalent non-equilibrium nanostructures remain elusive. Herein, we report synthetic polymer chains that consume a bivalent crosslinker in the presence of light to fold into a covalently bonded macromolecular architecture, namely a single chain nanoparticle (SCNP, Scheme 1a).^23–26^ Whereas the natural inspiration of SCNPs, *i.e.* the folding of peptides into proteins, strives towards the minimum of the energetic landscape to create defined 3D architectures,^27^ our current synthetic system folds away from its minimum energy. In the absence of light, these non-equilibrium SCNPs unfold back into linear polymer chains, enabling for the first time to gate reversible folding of synthetic polymer chains with light. Such a reversible folding was thus far only achieved through the addition of chemical triggers,^28,29^ change of solvent,^30,31^ pH^32^ and high thermal^33,34^ or mechanical energy,^35,36^ which limit areas of applications since it prevents covalently bonded SCNPs to be reversibly folded under mild conditions. Moreover, light-gated reversible folding remained unprecedented in the past, as photoreversible cycloadditions have shown to forfeit their reversibility in the confined environment of the polymer chain.^37–39^

## Results and discussion

The herein explored underlying visible light-fueled reaction is based on the reversible photoinduced cycloaddition of a triazolinedione (TAD) with naphthalene, which was first reported in 1984 (ref. 40) yet only recently applied in materials science to reversibly control the transition of a viscoelastic liquid into a cross-linked light-stabilized dynamic material (LSDM).^41^ To apply the photo-Diels–Alder reaction for the non-equilibrium folding of single polymer chains, two copolymers (P1 and P2, Scheme 1b) of methyl methacrylate (MMA) and naphthalene-containing comonomer were synthesized *via* reversible addition–fragmentation chain-transfer polymerization (RAFT) (Table 1).

**Table 1:** Synthesized polymers P1 and P2 with their respective *M*_n_, *Đ*, percentage of naphthalene units and crosslinking points

Polymer	*M*_n_ a/g mol^−1^	*Đ* a	% of naphthalene units^b^	Crosslinking points^c^
P1	8200	1.1	14	9
P2	11 200	1.2	12	11

a Obtained from THF-SEC data calibrated on PMMA standards.

b The monomer ratios were calculated from ^1^H NMR spectra in THF-*d*_8_ (Fig. S19 and S21).

c Crosslinking points calculated from ^1^H NMR spectra (ESI chapter 2).

Based on previous small molecule studies, a 2-carboxyl naphthalene derivative was used to introduce the aromatic side chains, as its electronic structure enables a rapid formation and close to quantitative cycloreversion of the corresponding TAD-adduct.^41^ To avoid undesired reactivity that might potentially occur between the residual dithiobenzoate RAFT group and the TAD moieties, the polymer end group was removed prior to the photochemical experiments (see ESI 4.2.3 and 4.2.4†).^42^ The resulting naphthalene-containing polymers were subsequently crosslinked using a bivalent 1,6-hexamethylene bistriazolinedione (BisTAD). It should be noted that an external crosslinker-mediated folding strategy was chosen over a hybrid TAD- and naphthalene-containing polymer, since TAD units on the polymer may degrade over time or undergo a competing light-induced polymerization.^43,44^ To investigate the SCNP folding, a mixture of P2 in dry, deoxygenized acetonitrile (*c* = 0.05 mg mL^−1^) and BisTAD (3 eq.) was irradiated with a green LED (10 W, *λ*_max_ = 525 nm, *E*_e_ = 60 mW cm^−2^). The course of the reaction during the irradiation process was monitored by UV/vis spectroscopy (Fig. S1†). The recorded spectra display the expected decrease in absorption during irradiation, resulting from the change in aromaticity of the naphthalene unit (Scheme 1b) as well as the consumption of the chromophoric azobond of the BisTAD moiety.^41^ However, since the absorption of both species overlaps in the UV region, the end point of the cycloaddition is not immediately evident. Indeed, whereas the absorption of the BisTAD moiety is prominent between *λ* = 450–550 nm, its decrease throughout the period of irradiation only confirms TAD consumption, either arising from the desired cycloaddition or the occurrence of unwanted side reactions. Nonetheless, both BisTAD and naphthalene units show a strong absorption between *λ* = 240–400 nm (UV/vis spectra of both individual compounds are shown in Fig. S2†). The characteristic absorption pattern of the naphthalene moiety (*λ*_max_ = 280 nm) was hence monitored during irradiation, which reached a plateau after 10 min. Whereas an overlap in absorption of both chromophores prevents an exact quantification of the reaction progress, a decrease of the characteristic naphthalene bands indicates that naphthalene crosslinking points are consumed for the intramolecular reaction (Fig. S1 and S2†).^42^

**Fig. 1:**
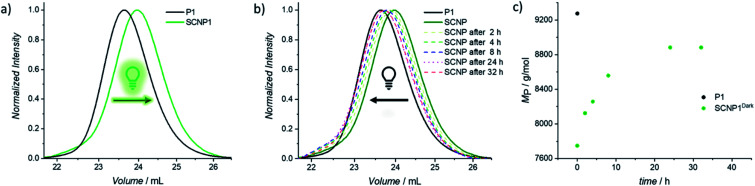
(a) Normalized UV-SEC-trace at 250 nm of the crosslinker mediated folding of P1 (black line) with BisTAD using green light (*λ*_max_ = 525 nm). The resulting collapsed polymer chain is displayed in dark green (SCNP1). (b) Normalized UV-SEC-traces at 250 nm of the stepwise unfolding process of SCNP1 over time in the dark. (c) Evolution of *M*_P_ during the stepwise unfolding process over time of SCNP1.

To confirm that the decrease in absorption also results in the desired macromolecular response of the system, size exclusion chromatography (SEC) traces were recorded as soon as possible after 20 min of irradiation in order to minimize the system's dark time instability (Fig. 1a). Compared to the parent polymer P1 (black solid line), the resulting SCNP1 (dark green solid line) has a significantly increased elution time, which results from a decrease in the hydrodynamic volume upon intramolecular crosslinking-induced compaction of the polymer chain.^42,45,46^ Along with the crosslinker-mediated folding, the apparent molecular weight of P1 decreased from *M*_n_ = 8200 g mol^−1^ to *M*_n_ = 7100 g mol^−1^. To verify the similar decrease in apparent molecular weight for P2 (*i.e. M*_n_ = 11 200 g mol^−1^ to *M*_n_ = 10 000 g mol^−1^, Fig. S7 and S8†), resulting from an absolute decrease in hydrodynamic diameter, diffusion ordered nuclear magnetic resonance spectroscopy (DOSY-NMR) measurements were carried out. The hydrodynamic diameter (*D*_H_) of the parent polymer P2 (*D*_H_ = 5.50 nm) indeed decreased by 11% upon single chain folding into SCNP2 (*D*_H_ = 4.92 nm). Furthermore, offline ^1^H NMR of SCNP2 verified that the observed contraction can be attributed to the transformation of the naphthalene side chain moieties into their corresponding TAD/naphthalene cycloadducts, with approximately 45% of naphthalene consumption reached 2 h after irradiation (*δ* = 8.75–8.50 ppm, Fig. S23–S26†).

To achieve a maximum contraction of SCNP2, the equivalents of crosslinker were found to be a key parameter. Indeed, when less than 3 eq. of BisTAD are used for the folding of P2, the obtained single-chain nanoparticle did not reach its maximum folded state (Fig. S10,† for 1 and 2 eq. BisTAD). It should be noted that the use of such super-stoichiometric amounts is a well-known requirement to achieve external crosslinker-mediated SCNP folding.^47^ When the amount of BisTAD exceeded 3 eq., however, intermolecular crosslinking of the polymer strands became apparent by the notable shoulder in the SEC traces at shorter elution times (Fig. S10,† for 6 and 9 eq. BisTAD). The observed intermolecular crosslinking can be rationalized by a higher propensity of TAD–TAD homo-polymerization with increased TAD concentration and a larger number of monovalently bound crosslinker moieties that can undergo intermolecular polymer reactions.

Next to the crosslinker equivalents, it is well-known that the solvent choice can also have a pronounced influence on the SCNP formation.^48^ To investigate the solvent effect, identical folding procedures were also carried out in dichloromethane, ethyl acetate and hexane. In all cases, no change in elution time was observed from SEC analysis (Fig. S11†), although upon folding P2 in dichloromethane, a second polymer distribution was noted at shorter elution times, thus indicating an expansion rather than the expected compaction. Thus, unlike acetonitrile, dichloromethane was identified as a non-preferred solvent for SCNP formation, which is likely attributed to its ability to promote TAD-homo-polymerization upon irradiation.^44^ The solvent choice thus has to meet a fine balance between polarity to dissolve both the polymer and TAD crosslinker and suppressing competing TAD photopolymerization.

To investigate the dissipative unfolding of SCNP1, the reaction solution was deprived of its fuel for 32 h at ambient temperature by placing it in the dark and the kinetics of the unfolding process were examined. In the absence of light, the macromolecular architecture is expected to unfold since the spontaneous thermal cycloreversion of the TAD/naphthalene adduct would not be compensated for by the light-induced cycloaddition reaction (as was assessed for the cycloaddition/cycloreversion of naphthalene derivative N1 and 4-*n*-butyl-TAD, Fig. S31†). To reach its initial conformation, the SCNP must thus proceed through several consecutive unfolding steps – each representing one covalent bond cleavage. The unfolding process was followed *via* SEC measurements after different time intervals in the absence of light (Fig. 1b). In comparison to the collapsed SCNP1 (dark green solid line), the SEC traces of the resulting SCNP1 after 2, 4, 8 and 24 h of darkness (dashed line, see Fig. 1b) show a significant decrease in elution time. The apparent *M*_P_ increases with increasing time in the absence of light, thereby indicating the unfolding of the macromolecular architecture over time. Specifically, the process of unfolding is rapid in the beginning, yet slows down over time, with the *M*_P_ increasing by 4.2% in the first 2 h compared to only an additional 3.5% between 8 and 24 h (Fig. 1c). After 24 h, a plateau *M*_P_ value is reached, at 96% recovery of the starting polymer.

To further assess the unfolding of the non-equilibrium SCNPs, DOSY-NMR was employed, revealing a hydrodynamic diameter for SCNP2^24 h-Dark^ of *D*_H_ = 5.36 nm. Thus, the polymer chain unfolds to 97% of the initial parent polymer diameter within 24 h. As observed from the ^1^H NMR of SCNP2 recorded after 24 h of standing in the dark, 96% of free naphthalene is present (*δ* = 8.75–8.50 ppm, Fig. S23†), whereas 3% of cycloadduct is still present, which might account for the residual contraction of the polymer. Further analysis of the unfolding process *via* UV/vis spectroscopy displayed an increased absorption in the naphthalene area at 280 nm over time (Fig. S3†). The starting intensity was, however, not fully recovered in our experiments, although this can be attributed to a slow degradation of the excess of BisTAD crosslinker over such a long time scale.^49,50^ Such irreversible TAD degradation was indeed observed when a plain solution of the BisTAD was irradiated in the absence of P1 or P2 at the concentrations used for the folding process (0.025 mg mL^−1^, irradiation for nearly 4 h, monitored *via* UV/vis absorption, see Fig. S4†).

Following the single chain polymer compaction upon irradiation, the macromolecule is thus shown to spontaneously unfold when left in the dark. The close to complete unfolding of the SCNPs can be rationalized by the dynamic nature of the TAD/naphthalene bond within the closed chemical system. The TAD/naphthalene cycloaddition reaches an equilibrium state in the dark, which strongly favors the starting reagents in the case of unconfined small molecules of naphthalene and TAD.^41^ Because of the high local concentrations within the confined environment of the polymer coil and a less pronounced loss of entropic contribution upon addition compared to non-polymer bound molecules, the equilibrium is apparently shifted towards the cycloadduct, resulting in a slightly folded SCNP1^24 h-Dark^ with regard to its parent polymer P1. A notable small degree of TAD/naphthalene cycloaddition occurring in the dark was also observed, as evidenced by the slight increase of elution time of P1 when stored with BisTAD overnight without being subjected to irradiation (Fig. S9†).

**Fig. 2:**
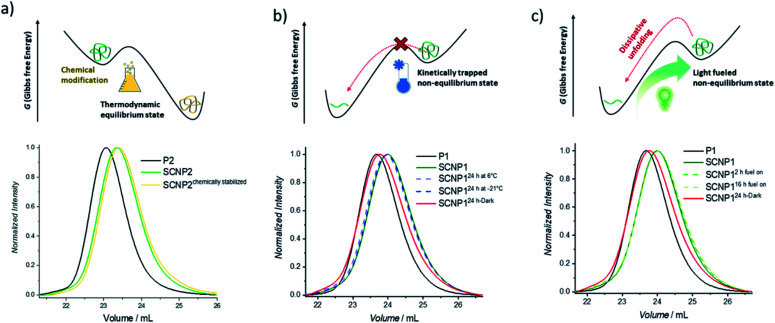
Schematic display of hypothetic energy landscapes (top) and normalized UV-SEC traces (bottom) of the (a) kinetically trapped non-equilibrium macromolecular architecture, (b) chemically stabilized folded macromolecular architecture, and (c) light-fueled stabilization of the non-equilibrium macromolecular architecture.

After having established that the single polymer chain can be pushed from its equilibrium into a higher energetic folded conformation using green light as the fuel, it was next explored how the folded conformation can be sustained. Firstly, the ability to modulate the energetic landscape to make the folded state thermodynamically favored was investigated (Fig. 2a). If the double bond generated by the cycloaddition is removed under reductive conditions, the cycloreversion can no longer take place and the folded architecture should become more thermodynamically favored.^41,51^ To remove the double bond and hence chemically stabilize the SCNP, P2 was subjected to a diimide-mediated reduction after being folded (see ESI section 4.2.6†). Thus, the SCNP can be trapped at the maximum folded state, resulting in a longer elution time than the SCNP2 obtained directly after irradiation, which already unfolds during the SEC run.

To maintain the folded architecture without chemical modification, low temperatures were investigated to kinetically trap the non-equilibrium state by lowering the available energy for the system below the activation barrier required to allow for the cycloreversion to become feasible (Fig. 2b). When the collapsed SCNP1 is stored well below ambient temperature, the unfolding rate is indeed drastically reduced. Specifically, storage for 24 h at −21 °C (dark blue dashed line) results in an identical SEC elugram compared to SCNP1 immediately measured after irradiation. In contrast, after 24 h storage at 6 °C, a slight shift towards higher *M*_n_ is visible, which indicates the slow unfolding of the architecture (light blue dashed line). These observations demonstrate the kinetic trapping of the folded architecture and indicate that low temperatures provide a suitably high thermal barrier to halt the cycloreversion and thus SCNP unfolding.

Finally, the fuel driven equilibrium state was investigated. To stabilize SCNP1, formed upon 30 min green light irradiation of in the presence of BisTAD, the reaction mixture containing SCNP1 was continuously subjected to light and SEC traces were recorded after 2 h and 16 h of extended irradiation, respectively. The obtained traces (Fig. 2c, green dashed lines) display the same elution times and demonstrate that the macromolecular architecture can be kept in the non-equilibrium state as long as fuel is provided. In contrast, leaving SCNP1 – obtained after 30 min of irradiation – standing in the dark for 24 h results in an increase of the hydrodynamic volume back to the equilibrium of the freely dissolved polymer (Fig. 2c, red solid line).

**Fig. 3:**
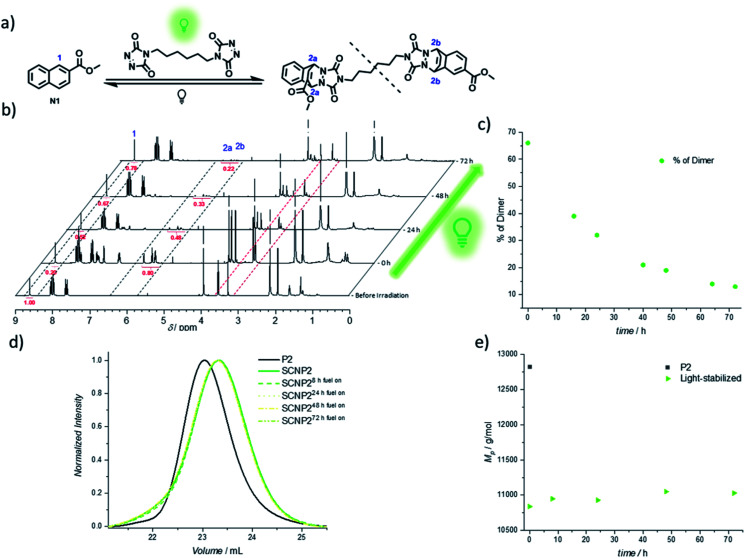
Analysis of long-term light-fueled stabilization of TAD/naphthalene cycloadducts. (a) Small molecule reaction equation with naphthalene N1. The dashed line displays a break since different regio-isomers are formed. (b) ^1^H NMR analysis (in CD_3_CN) of the light-stabilization on small molecule scale with continuous irradiation with NMR spectra displayed before irradiation, after the first irradiation (0 h) and at 24, 48 and 72 h of irradiation. The dashed marked areas display the naphthalene signal 1 (starting material), bridge protons 2a and 2b (cycloadducts) and aliphatic TAD-signals 3. (c) Plot of percentage of cycloadduct present in the solution at different times. (d) Normalized UV-SEC traces (at 250 nm) measurements after 8, 24, 48 and 72 h of light-fueled stabilization of SCNP2. (e) Evolution of *M*_P_ during the light stabilization of SCNP2.

The continued light irradiation thus provides the necessary driving force to maintain the photostationary state, yielding the thermally unstable cycloadduct. An important consideration, however, is that even in the presence of green light, the cycloadduct is expected to constantly cleave and reform over short time intervals during the fuel-driven process, although with the equilibrium strongly favoring the cycloadduct side (as verified with an exchange experiment during irradiation, see Fig. S32†). Since prolonged irradiation of freely existing TAD moieties can also promote undesired transformations such as photodegradation or photopolymerization, it is essential to further probe the limits of the light-fueled reaction over much longer time frames. Therefore, a naphthalene derivative N1 was synthesized, which mimics the used naphthalene monomer and hence allows for small molecule investigations concerning the TAD-based photocycloaddition (Fig. 3a). The irradiation of N1 in the presence of BisTAD (0.4 eq.) was conducted at a relatively high concentration (3.6 mg mL^−1^) in deuterated deoxygenated acetonitrile in order to monitor the cycloadduct conversion *via*^1^H NMR (Fig. 3a, note that different regioisomers are formed, see ESI 4.2.7†). After 40 min of irradiation, complete BisTAD consumption can be observed from the ^1^H NMR spectrum (*δ* = 3.6 ppm), indicating quantitative formation of the cycloadduct (Fig. 3b, 0 h after irradiation). This marked the starting point of the prolonged irradiation experiment, where the percentage of intact cycloadduct decreased significantly with percentages as low as 28% reached after 72 h (compared to 100% cycloadduct at the start of prolonged irradiation, see Fig. 3c). In contrast to the model study, the light-stabilization of the single polymer chain was much more reliable over the course of 72 h of irradiation. Indeed, as long as fuel could be consumed, the non-equilibrium architecture was maintained, enabling its stabilization for up to three days (Fig. 3d and e).

The confined multivalent environment of the SCNP structure thus appears to be key for the efficient light-stabilization. Indeed, for the non-confined small molecule, the TAD and naphthalene moiety are expected to diffuse apart more readily after cycloreversion. Upon subsequent photoexcitation, the TAD/naphthalene reaction therefore will have to compete to a larger extent with de-excitation processes or TAD-based side reactions such as polymerization and photodegradation, resulting in a loss fraction of cycloadduct. On the other hand, in the confined environment of the SCNP, released TAD moieties are more likely to remain in close proximity to either their previous naphthalene binding partner or a number of other available pending naphthalene moieties, which is believed to facilitate the (re)formation of the desired TAD/naphthalene cycloadduct in the presence of light. Unlike purely photochemically gated systems based on photoreversible cycloadditions – where the macromolecular confinement favours the folding reaction to the point where unfolding becomes impossible^37–39,52^ – it herein appears that the combination of photochemical folding and thermal unfolding is ideal to execute these reactions selectively.

**Fig. 4:**
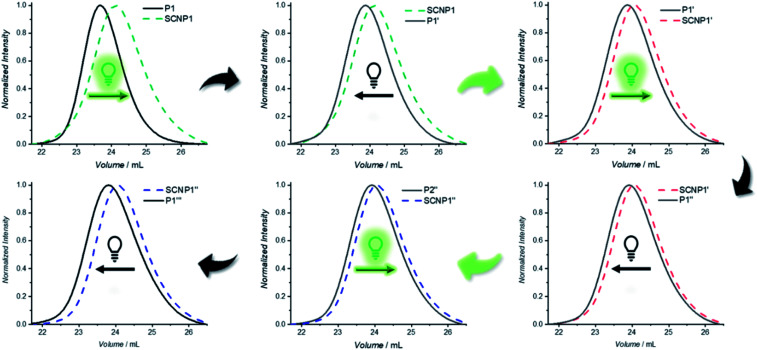
Reversible folding and unfolding of the macromolecular architecture. In a closed system, P1 (black line) was folded to SCNP1 (green dashed line, 20% contraction) upon 20 min of irradiation (525 nm). After 24 h SCNP1 unfolds to P1′ (green solid line), which was folded again, resulting in SCNP1′ (red dashed line, 9% folding). This unfolds back to P1′′ (solid red line), which can be folded once more into SCNP1′′ (blue dashed line, 7% folding). In the last step, SCNP1′′ unfolds again to form P1′′ (blue solid line).

One advantage of dynamic non-equilibrium systems is the inherent reversibility of the system. It is thus possible to undergo several folding and unfolding steps in a closed system with the same starting polymer. To investigate repetitive polymer compaction/unraveling, P1 was irradiated with BisTAD (3 eq.) for 20 min to obtain SCNP1 (green dashed line, compaction of 20%). The unfolded P1′, obtained after keeping SCNP1 24 h in the dark, displays a clear decrease in elution time. Following the first cycle, the solution was again irradiated for 20 min, resulting in a collapsed SCNP1′ (red dashed line, compaction of 9%) that subsequently unfolded after 24 h in the dark to P1′′. Finally, P1′′ was again irradiated for 20 min to obtain SCNP1′′ (blue dashed line, compaction of 7%), whereas P1′′ was retrieved after leaving SCNP1′′ unfold for 24 h in the dark (Fig. 4). Throughout the three consecutive folding/unfolding cycles, a significant change in the volume of the macromolecular architecture occurs during each cycle. To elucidate the reason for the hysteresis, it was investigated whether the naphthalene crosslinking points are no longer accessible because of irreversible side reactions, or whether the BisTAD crosslinker availability depletes as a result of its limited stability when being stored in solution for several days at ambient temperature. In addition, also photodegradation of the crosslinker could cause the noted hysteresis.

**Fig. 5:**
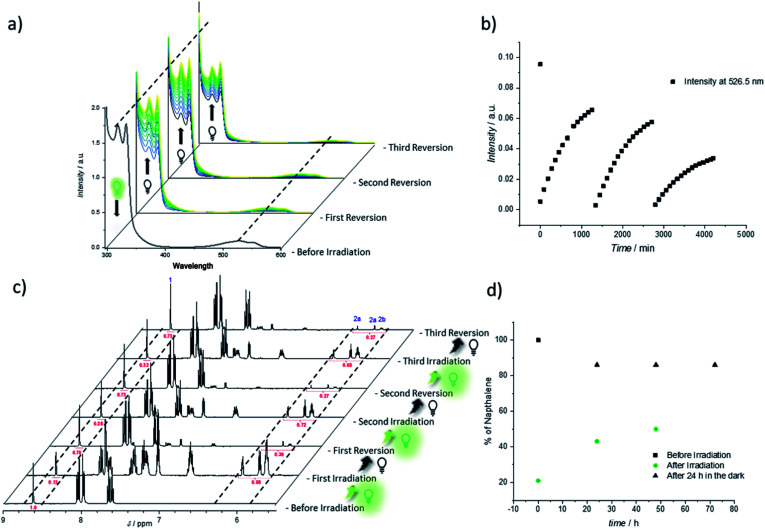
Reversible folding studies on small molecule scale using naphthalene N1. (a) UV/vis-spectra before and after three consecutive irradiation cycles (reversion in the dark). (b) Evolution of the UV/vis absorbance as a function of time at 526.5 nm (*λ*_max_ TAD absorption) over the three irradiation and reversion cycles. (c) ^1^H NMR analysis recorded during the irradiation cycles in CD_3_CN. Measurements were carried out before irradiation, after irradiation and after being kept 24 h in the dark, respectively. The dashed lines highlight the evolution of representative resonances, including naphthalene proton 1 (starting material) and the bridge protons 2a and 2b (cycloadduct). (d) Plot of the percentage of naphthalene N1 present in the sample directly after irradiation and after 24 h of cycloreversion in the dark.

Thus, the repetitive TAD/naphthalene cycloaddition/reversion was investigated by means of small molecule studies on a closed system of N1 (3.6 mg mL^−1^) in acetonitrile (0.7 mL) containing a substoichiometric amount of BisTAD (0.4 eq.). The resulting mixture was deoxygenated and then irradiated for 40 min. For three consecutive cycles, UV/vis (Fig. 5a and b) as well as ^1^H NMR (Fig. 5c and d) were measured before and after irradiation, as well as after 24 h upon standing in the dark. In the resulting UV/vis spectra (Fig. 5a) the characteristic TAD absorption band at 520–530 nm increases again after disappearance following every irradiation. The recovered signal results from the liberation of free TAD moieties after every irradiation. Nevertheless, a hysteresis is visible, hence resulting in a decrease of maximum intensity after every cycle (Fig. 5a and b highlighted for 320 nm and 520 nm). Reversible cycloadduct formation was also observed with H-NMR, were the conversion was followed *via* the appearance and disappearance of the bridge-protons of the Diels–Alder product (Fig. 5c, bridge protons 2a and 2b, 6.2–5.8 ppm). After 24 h in the dark, close to 84% of naphthalene N1 was restored at the end of each cycle. Following each irradiation step, the amount of unreacted naphthalene increased (Fig. 5d), and therefore the amount of formed cycloadduct decreases, although the observed photobleaching after each irradiation step suggests the complete consumption of BisTAD (Fig. S29†). This is a first indication that the hysteresis results from TAD depletion, rather than from irreversible degradation of the naphthalene or cycloadduct. Remarkably, even after four days following the last reversion step, still 8% of cycloadduct was present in the reaction mixture (Fig. S30†). In fact, this obtained equilibrium point observed on a small molecule scale explains why the folded polymer chains did not entirely reform the starting polymers P1 and P2 after the fuel was removed (*cf.*Fig. 1b, 3a, b and 4).

To further investigate the source of hysteresis in the reversible process of each small molecule reversion, mass spectrometry was used to analyze the SCNP and small molecule reaction solutions. Thereby, 11 plausible TAD-based side-products were detected (Fig. S13 and Table S1†).^53,54^ When the UV/vis experiment was repeated at significantly lower concentrations for a better comparison with the concentration regime of SCNP folding, an even more pronounced change in the reversibility can be observed (concentrations 4 times higher than on SCNP scale, Fig. S5 and S6†). Whereas after irradiation the aborption intensity of the TAD moiety decreases similarly to the previous results (*λ* = 526.5 nm, Fig. S6†), the following reversion cycle recovers less than 50% of the initial intensity. The yield of regenerated TAD after subsequent reversion cycles decreases even further. These findings are in agreement with the result obtained for the SCNP folding where also very little recovery was visible in the UV/vis (Fig. S3†). In other words, these results indicate that the observed folding/unfolding hysteresis results from degradation of released BisTAD, while the polymer bound naphthalene moieties stay intact.

The diminished reversibility of the folding process can readily be overcome through the here exploited polymer design based on an external crosslinker mediated SCNP folding strategy. Indeed, additional TAD can readily be introduced into the system at any time to compensate for TAD depletion and hence re-instate the complete folding of the naphthalene-containing polymers. Thus, when additional BisTAD was added in a separate system after every unfolding (Fig. S12†), no significant change in elution time of the SCNP after the second folding cycle was observed (compaction of 10%). In contrast, when additional BisTAD was added before the third folding step into SCNP1′′ – which showed the most pronounced hysteresis in the case of P1′′ to SCNP1′′ – a notable increase in elution time was noted as compared to the SEC elugram obtained without the addition of new BisTAD (contraction of 11%, *i.e.* 4% more than without new TAD aliquots). Reversibility of the developed SCNP system can thus still be obtained, albeit not only upon consumption of light as a fuel, but also BisTAD over the course of time.

## Conclusions

We report an unprecedented light-fueled single polymer chain folding resulting in a covalently bound 3D macromolecular architecture existing in a non-equilibrium state. The system is based on two MMA/naphthalene copolymers (P1 and P2) synthesized using RAFT polymerization. In the presence of BisTAD as a crosslinker, both polymer chains can be folded (to SCNP1 and SCNP2) using green light (525 nm). In the absence of fuel at ambient temperature, the folded architecture unfolds, thereby reforming the free, random polymer coil as confirmed by SEC and DOSY-NMR measurements.

Furthermore, it was possible to sustain the folded architecture using three different gateways. A consistent fuel supply enables an intact SCNP1 over 3 days. In addition, the macromolecular architecture can be kinetically trapped using temperature as a tool. At temperatures below 6 °C, the cycloreversion between TAD and naphthalene becomes less viable and traps the folded architecture. Whereas these methods allow to maintain the SCNP in a non-equilibrium state, the folded structure was also brought to a new thermodynamic minimum by a chemical modification that halts the cycloreversion.

In a closed system, it was possible to repeat the folding/unfolding process three times, albeit with a small hysteresis that was attributed to a depleted BisTAD availability. Upon addition of new BisTAD to the closed system, however, the hysteresis was overcome. The herein introduced non-equilibrium SCNPs are believed to open the field of fuel-driven single polymer chain folding, which could mark an important step in imparting life inspired properties into synthetic polymers.

## Conflicts of interest

The authors declare no conflict of interests.

## Supplementary Material

SC-012-D0SC05818A-s001
