# Supplementary material for: Light-fueled dynamic covalent crosslinking of single polymer chains in non-equilibrium states
Source: Chem Sci. 2020 Nov 17;12(4):1302–10. doi: 10.1039/d0sc05818a (PMC8179028; doi:10.1039/d0sc05818a)
Supplement: SC-012-D0SC05818A-s001 [file SC-012-D0SC05818A-s001.pdf]

Electronic Supplementary Material (ESI) for Chemical Science.
This journal is © The Royal Society of Chemistry 2020

Supporting Information

Light-Fueled Dynamic Covalent Crosslinking of Single Polymer Chains in Non-Equilibrium States

Daniel Kodura, Hannes A. Houck, Fabian R. Bloesser, Anja S. Goldmann, Filip E. Du Prez, Hendrik Frisch and Christopher Barner-Kowollik

Contents

1. Additional experiments 3

1.1 UV/Vis Spectra 3

1.1.1 Reversible cycloaddition at low concentration 5

1.2. Additional SEC traces 7

1.3 Reversible folding with addition of new BisTAD 10

1.4 ESI-HR-MS analysis of plausible TAD-derived side products 11

2. Calculation of M1 per polymer chain 13

2.1 Calculation of M1 per polymer chain of P1 13

2.1 Calculation of M1 per polymer chain of P2 13

3. Experimental details 15

3.1 THF-SEC 15

3.2 1D NMR Measurements 15

3.3 Diffusion Ordered Spectroscopy (DOSY) NMR 15

3.4 UV-VIS Spectroscopy 16

3.5 LC-MS Analysis 16

3.6 Irradiation experiments with green light (λ_{max} = 525 nm): 16

4. Synthetic Procedures 18

4.1 Materials 18

4.2 Synthesis 18

4.2.1 Naphthalene Monomer (M1) 18

4.2.2 Synthesis of methyl 2-naphthoate (N1) 20

4.2.3 Synthesis of P1 21

4.2.4 Synthesis of P2 23

4.2.5 General procedure for the folding of P1 with BisTAD 25

4.2.6 General procedure for the folding of P2 with BisTAD 25

4.2.7 Procedure for the light stabilization of naphthalene N1 29

4.2.8 Procedure for ^{1}H-NMR measurements of reversible cycloaddition/cycloreversion of naphthalene N1 with BisTAD 31

4.2.9 Procedure for cycloaddition/cycloreversion exchange experiment under continuous irradiation 35

4.2.10 General procedure for light-fueled stabilization of P2. 37

4.2.11 Procedure for UV/Vis measurements of reversible cycloaddition/cycloreversion of naphthalene N1 37

4.2.12 General procedure for chemical SCNP stabilization upon reduction 38
4.2.13 General procedure for light-fueled stabilization of P1. 38
4.2.14 General procedure for the kinetic trapping of SCNPs below ambient temperature 38
5. References 39
6. Primary DOSY Data – Appendix 39

1. Additional experiments

1.1 UV/Vis Spectra

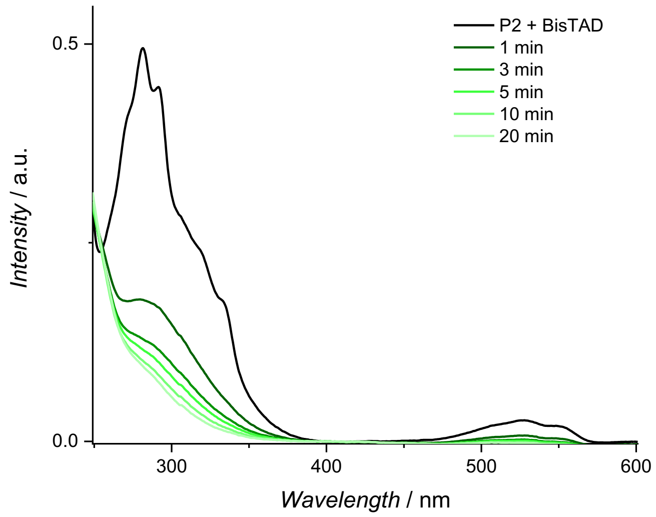

Figure S1: UV/Vis spectra recorded during the irradiation process of **P2** (c = 0.05 mg mL^{-1}) and **BisTAD** (c = 0.02 mg mL^{-1}) with a green LED (10 W, λ_{max}= 525 nm) in acetonitrile

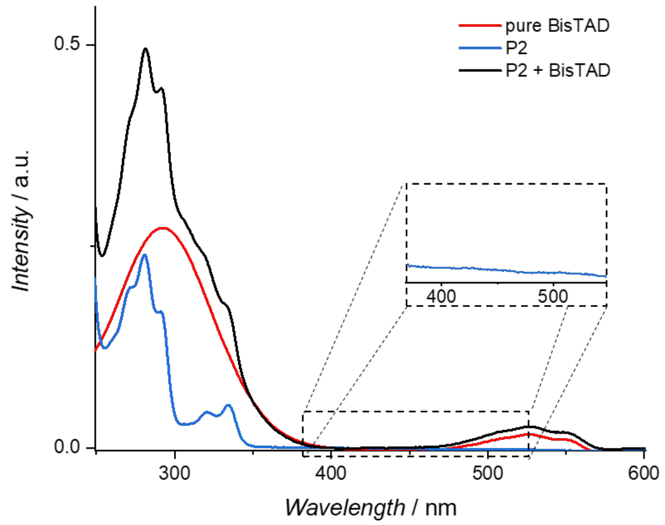

Figure S2: UV/Vis spectrum of **P2** (0.05 mg mL^{-1}, blue), **BisTAD** (0.02 mg mL^{-1}, red) and the reaction mixture of **P2** with 3 eq. **BisTAD** (black) in acetonitrile. The inset between 400-500 nm provides an indication of the successful removal of the RAFT-end-group.

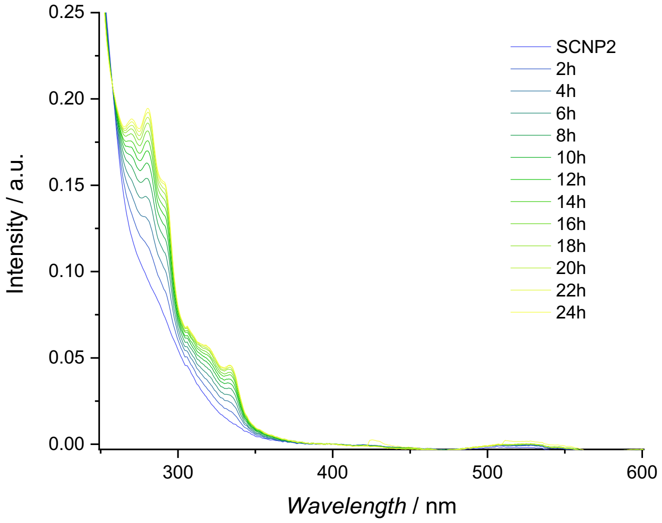

Figure S3: UV/Vis spectra recorded during the unfolding process of **SCNP2** in acetonitrile (c = 0.05 mg mL^{-1}). Note that a quantification of the naphthalene consumption and recovery is not possible through UV/Vis spectroscopy, since TAD absorbs significantly in the same region (compare Figure S2) and is prone to a variety of degradation reactions (compare chapter 1.4). While the naphthalene recovery increases absorption, the solvent impurity dependent TAD degradation of both released and excess TAD decreases the absorption in this region.

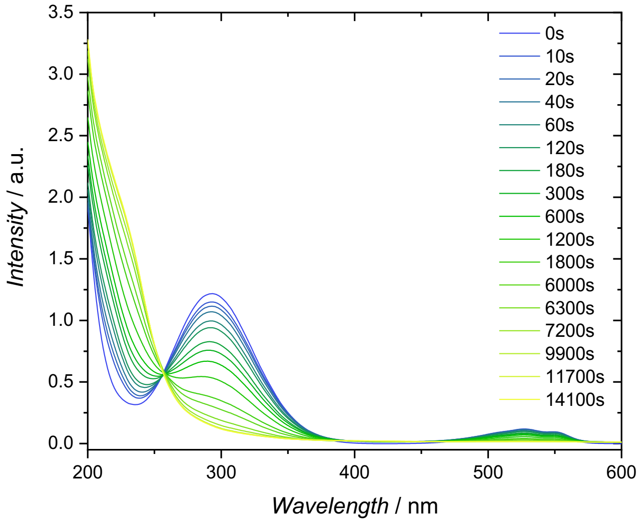

Figure S4: UV/Vis spectra recorded during the irradiation process of pure **BisTAD** with a green LED (10 W, $\lambda_{\max}$ = 525 nm) in acetonitrile.

1.1.1 Reversible cycloaddition at low concentration

The influence of concentration on the reversibility of the TAD/naphthalene cycloaddition/cycloreversion is not negligible. Whereas higher concentrations of the low molecular weight derivatives **N1** and **BisTAD** lead to a good reversibility and a consistent hysteresis, lowering the concentration (to 0.2 mg mL^{-1}) resulted in significant lower amounts of cycloadduct being formed with more TAD consumed in side reaction, thus resulting in a lower extent of reversibility. This is indeed observed from the UV/vis spectra depicted in Figures S5 and S6, which indicate only a minor fraction of TAD being regenerated at the end of each cycle.

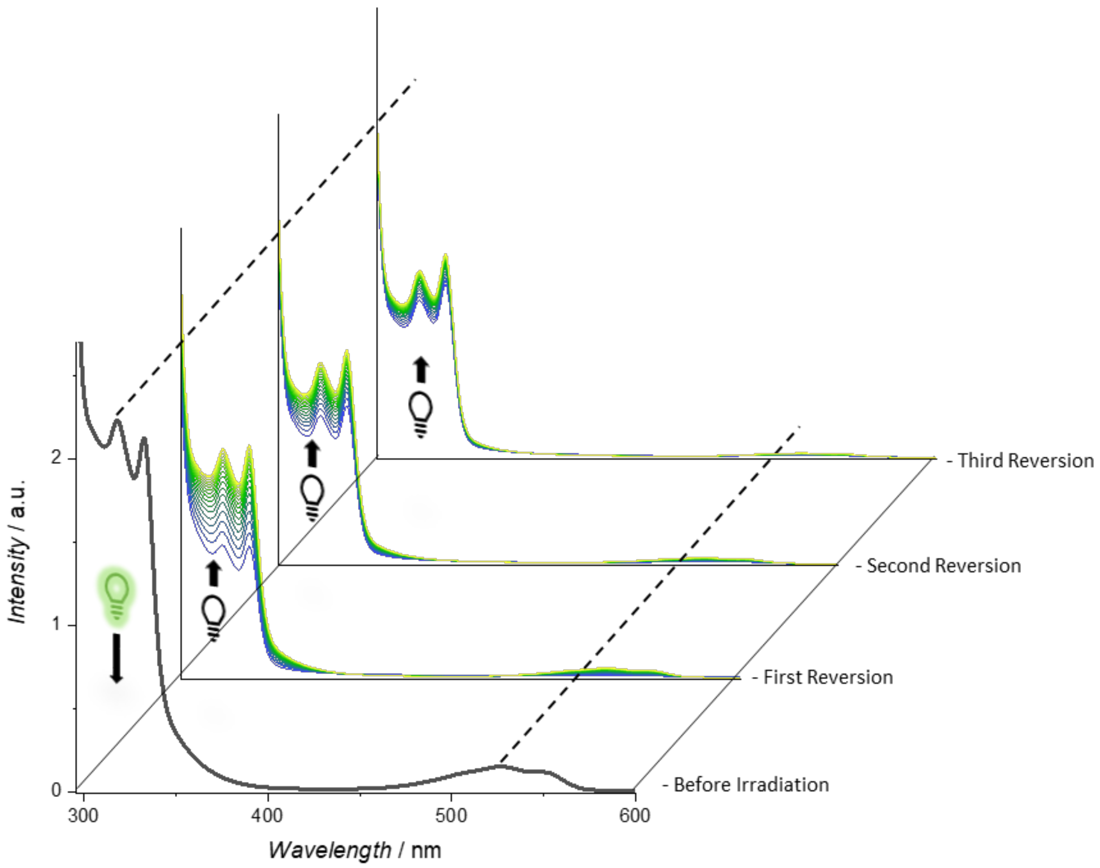

Figure S5: UV/Vis spectra recorded during the cycloreversion process of naphthalene **N1** and **BisTAD** with concentrations four times higher than on the SCNP scale (c = 0.2 mg mL^{-1}). Compared to the same experiments with an 18-fold higher concentration (cf. Figure 2, main paper, c = 3.6 mg mL^{-1}), the reversibility of the TAD/naphthalene system is considerably lower.

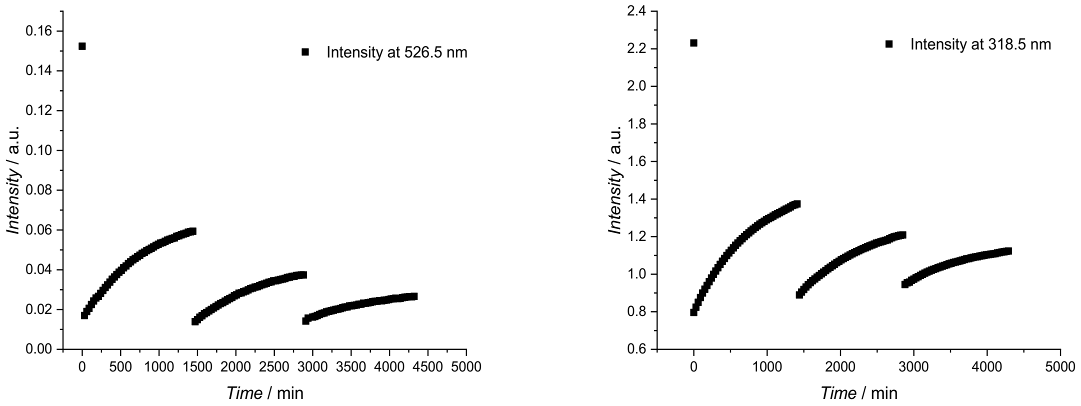

Figure S6: Plot of UV/Vis absorbance over three consecutive irradiation and reversion cycles, derived from Figure S5. Left absorbance intensity at λ = 526.5 nm, right at λ = 318.5 nm

1.2. Additional SEC traces

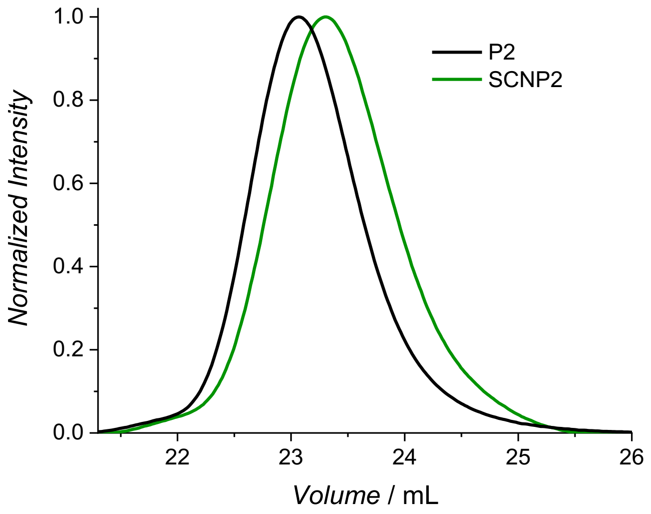

Figure S7: Normalized UV-THF-SEC trace at 250 nm of **P2** (back) and resulting **SCNP2** (green), formed after green light irradiation in the presence of 3 eq. **BisTAD**.

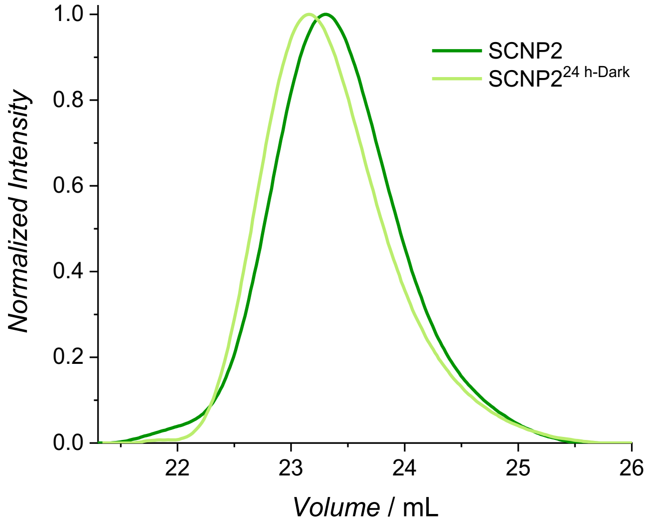

Figure S8: Normalized UV-THF-SEC trace at 250 nm of **SCNP2** (dark green) and the resulting **SCNP2**$^{\mathbf{(24\ h\text{-}Dark)}}$, obtained after standing in the dark for 24 h at ambient temperature (light green).

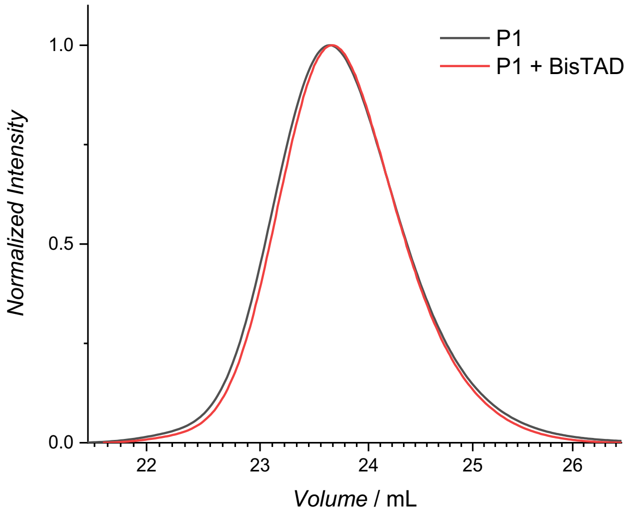

Figure S9: Normalized UV-THF-SEC trace at 250 nm of **P1** (back) and a mixture of **P1** with **BisTAD** in acetonitrile, stored for 24 h in the dark (red).

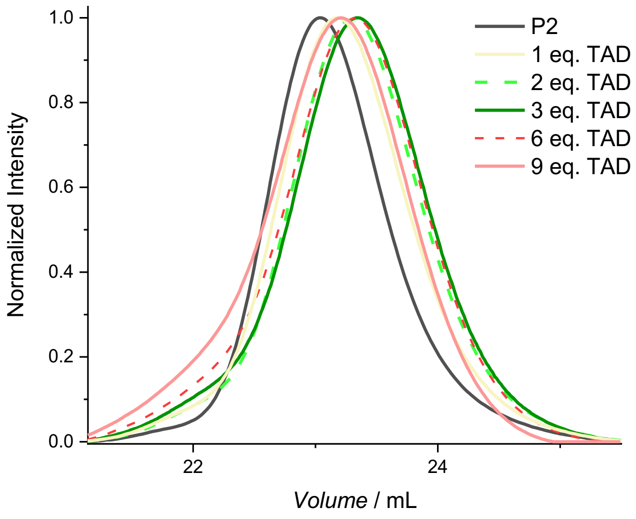

Figure S10: Normalized UV-THF-SEC trace at 250 nm of **P2** (back) and folded **SCNP2**, obtained upon green light irradiation of **P2** with different equivalents of **BisTAD**. Whereas with increasing amount of crosslinker (i.e. from 1 eq. to 3 eq.), an increase in elution time is evident, further increasing the amount of crosslinker (i.e. to 6 and 9 eq. **BisTAD**) apparently resulted in more inter-chain crosslinking, as evidenced from a broader dispersity and a more pronounced shoulder at shorter elution times.

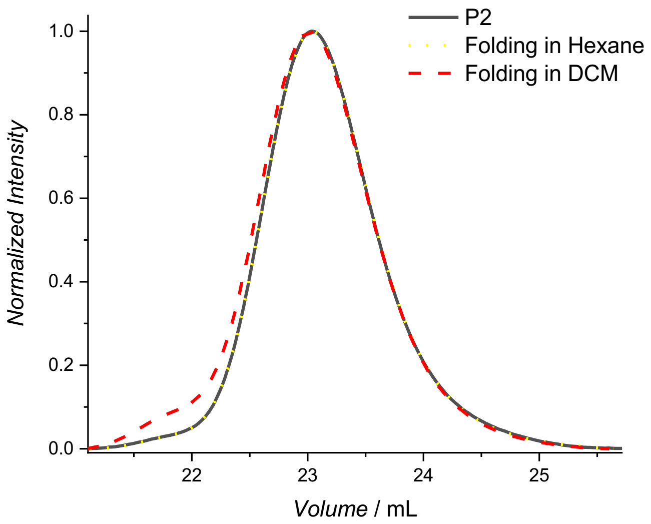

Figure S11: Normalized UV-THF-SEC trace at 250 nm of **P2** (back) and folded **SCNP2** with dichloromethane (DCM) and hexane as solvent. While DCM leads to a small increase in molecular weight, hexane and ethyl acetate as a solvent appear not to effect the folding process.

1.3 Reversible folding with addition of new BisTAD

As shown in **Figure 4** in the main paper, the SCNP can be reversibly folded and unfolded with a slight hysteresis throughout the process. To control whether the hysteresis occurs because of the formation of side products on the polymer or as a result of TAD degradation, additional experiments were carried out. Specifically, the reaction mixture of **P1’** (1 mL, prepared as described in section 4.2.5) was treated with 1 eq. of new **BisTAD** (0.025 mg). After purging with argon, the solution was irradiated for 20 min, the solvent was removed and subjected to SEC measurement. The same procedure was applied to the reaction mixture of **P1’’**. Whereas for **SCNP1’ with new BisTAD** no increase in elution time is observed, the resulting **SCNP1’’ with new BisTAD** has a significantly higher elution time, indicating a higher contraction and thus evidencing that the hysteresis most likely can be attributed to TAD-degradation occuring over time.

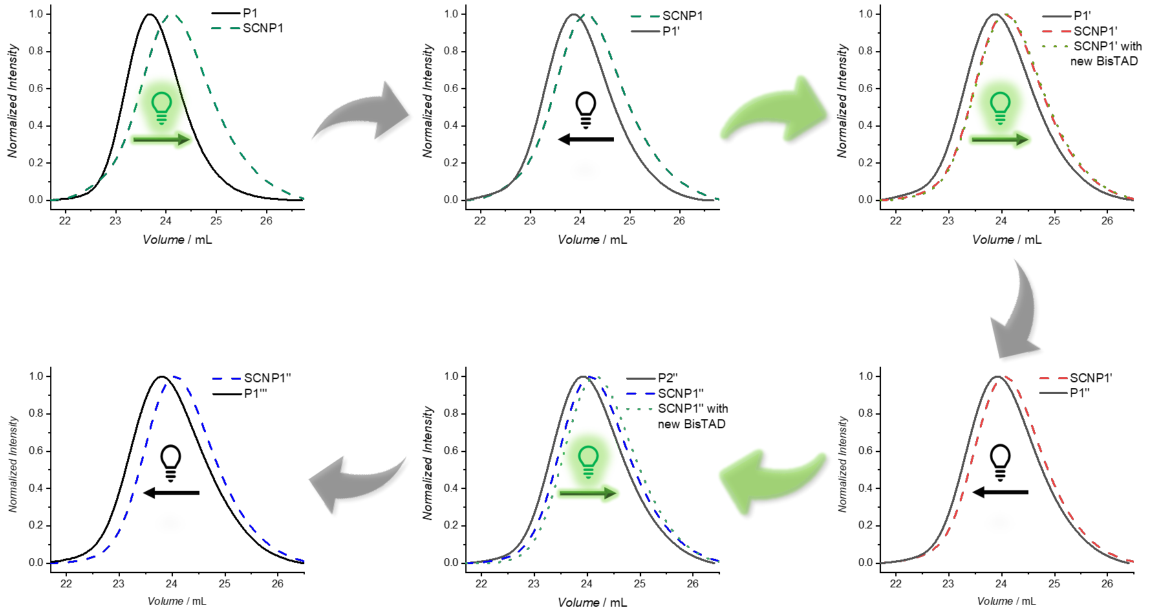

Figure S12: Reversible folding and unfolding of **P1** over several cycles in a closed system. **P1** (black line) was folded to **SCNP1** (green dashed line) upon 20 min of irradiation. After 24 h at ambient temperature, **SCNP1** unfolds to **P1’** (green solid line). Subsequently, **P1’** was irradiated again, resulting in **SCNP1’** (red dashed line). When additional **BisTAD** is added before the subsequent irradiation step, no notable change in elution time is observed (**SCNP1’ with new BisTAD**, green dotted line). **SCNP1’** unfolds again to **P1’’** (black line), which again can be folded into **SCNP1’’** (blue dashed line). If now additional **BisTAD** is added before irradiation, the resulting **SCNP1’’ with new BisTAD** (green dotted line) has a significantly higher elution time**.** In the last step, **SCNP1’’** unfolds again to form **P1’’’** (black line).

1.4 ESI-HR-MS analysis of plausible TAD-derived side products

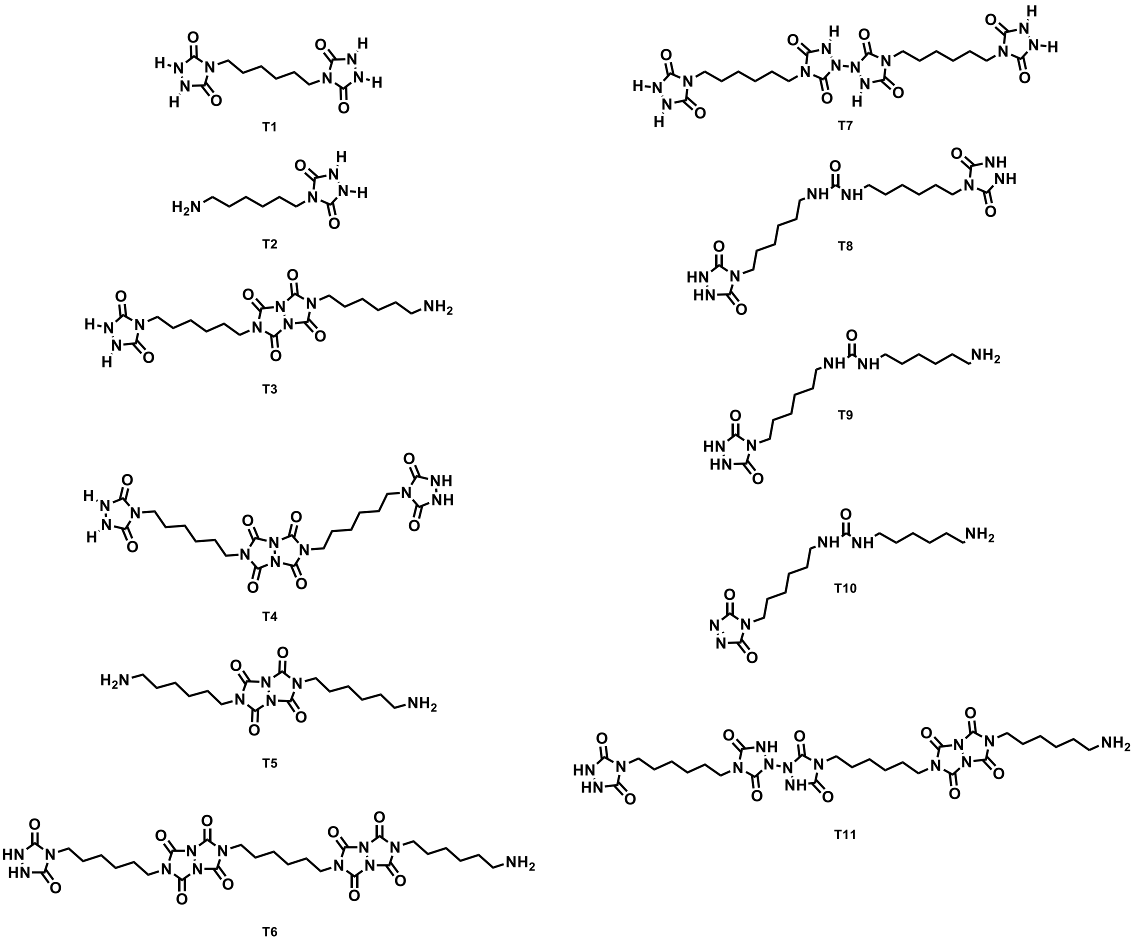

Figure S13: Chemical structures of plausible TAD-based side products generated through TAD homo-polymerization, hydrolysis (reduction to urazole and amine) or dimerization detected by HR-ESI-MS analysis, which might have formed during consecutive cycloaddition/cycloreversion cycles.

Table S1: Masses of plausible TAD-based side products (displayed in Figure S13), identified from the HR-ESI-MS spectrum.

TAD-based compound	$[M+H]^+$		$[M+Na]^+$		$[M+NH_4]^+$	
	calculated	measured	calculated	measured	calculated	measured
T1	285.1306	285.1313	307.1125	307.1133		
T2	201.1346	201.1351	223.1165	223.1170		
T3	453.2205	453.2216	475.2024	475.2035	470.2470	470.2480
T4	537.2164	537.2176	559.1984	559.1993	554.2430	554.2440
T5	369.2245	369.2255	391.2064	391.2074		
T6	705.3063	705.3075	727.2883	727.2885		
T7	789.3032	789.3039				
T8	427.2412	427.2421	449.2231	449.2240		
T9	343.2452	343.2458	363.2115	363.2113		
T10	341.2296	341.2304				
T11	735.3281	735.3284	757.3101	757.3105	752.3547	752.3543

2. Calculation of M1 per polymer chain

2.1 Calculation of M1 per polymer chain of P1

Using the percentage of **M1** in **P1** obtained from the ^{1}H-NMR spectra and the $\overline{M}_n$ obtained from the SEC, the average number of **M1** units and MMA monomers per polymer chain were calculated as follows:

$$n(M1) = n(monomers) \bullet X(M1)$$

$$n(MMA) = n(monomers) \bullet X(MMA)$$

With the average number of monomers per polymer chain $n(monomers)$:

$$n(monomers) = \frac{\overline{M}_n \ (Polymer)}{M_{Average}(monomer)}$$

With the average monomer mass ($M_{Average}$):

$$M_{Average} = X(M1) \bullet M(M1) + X(MMA) \bullet M(MMA)$$

Resulting in the following formula:

$$n(M1) = \left(\frac{\overline{M}_n \ (Polymer)}{X(M1) \bullet M(M1) + X(MMA) \bullet M(MMA)}\right) \bullet X(M1)$$

For **P1** this leads to:

$$n(M1) = \left(\frac{8{,}200 \ g\ mol^{-1}}{0.14 \bullet 284 \ g\ mol^{-1} + 0.86 \bullet 100 \ g\ mol^{-1}}\right) \bullet 0.14 = 9$$

$$n(MMA) = \left(\frac{8{,}200 \ g\ mol^{-1}}{0.14 \bullet 284 \ g\ mol^{-1} + 0.86 \bullet 100 \ g\ mol^{-1}}\right) \bullet 0.86 = 56$$

2.1 Calculation of M1 per polymer chain of P2

Using the percentage of **M1** in **P2** obtained from the ^{1}H-NMR spectra and the $\overline{M}_n$ obtained from the SEC, the average number of **M1** units and MMA monomers per polymer chain were calculated as follows:

$$n(M1) = n(monomers) \bullet X(M1)$$

$$n(MMA) = n(monomers) \bullet X(MMA)$$

With the average number of monomers per polymer chain $n(monomers)$:

$$n(monomers) = \frac{\overline{M}_n \ (Polymer)}{M_{Average}(monomer)}$$

With the average monomer mass ($M_{Average}$):

$$M_{Average} = X(M1) \bullet M(M1) + X(MMA) \bullet M(MMA)$$

Resulting in the following formula:

$$n(M1) = \left(\frac{\overline{M}_n \ (Polymer)}{X(M1) \bullet M(M1) + X(MMA) \bullet M(MMA)} \right) \bullet X(M1)$$

For **P2** this leads to:

$$n(M1) = \left(\frac{11{,}200 \ \ g \, mol^{-1}}{0.12 \bullet 284 \ \ g \, mol^{-1} + 0.88 \bullet 100 \ \ g \, mol^{-1}} \right) \bullet 0.12 = 11$$

$$n(MMA) = \left(\frac{11{,}200 \ \ g \, mol^{-1}}{0.12 \bullet 284 \ \ g \, mol^{-1} + 0.88 \bullet 100 \ \ g \, mol^{-1}} \right) \bullet 0.88 = 80$$

3. Experimental details

3.1 THF-SEC

All samples were measured in amber SEC-vials to prevent exposure to ambient light. The SEC measurements were conducted on a PSS SECurity2 system consisting of a PSS SECurity Degasser, PSS SECurity TCC6000 Column Oven (35 °C), PSS SDV Column Set (8 x 150 mm x 5 µm Precolumn, 8 x 300 mm 5 µm Analytical Columns, 100000 Å, 1000 Å and 100 Å) and an Agilent 1260 Infinity Isocratic Pump, Agilent 1260 Infinity Standard Autosampler, Agilent 1260 Infinity Diode Array and Multiple Wavelength Detector (A: 254 nm, B: 360 nm), Agilent 1260 Infinity Refractive Index Detector (35 °C). HPLC grade THF, stabilized with BHT, is used as eluent at a flow rate of 1 $mL{\cdot}min^{-1}$. Narrow disperse linear poly(methyl methacrylate) ($\overline{M}_n$: 202 $g{\cdot}mol^{-1}$ to $2.2{\cdot}10^6$ $g{\cdot}mol^{-1}$) standards (PSS ReadyCal) were used as calibrants. All samples were passed over 0.22 µm PTFE membrane filters. Molecular weight and dispersity analysis were performed in PSS WinGPC UniChrom software (version 8.2). To calculate the respective percentage of folding/unfolding, the following formula was used (*x* = *n* or *p*).

$$Contraction/Expansion_{\%} = 100 - \frac{M_{x,new}}{M_{x,old}} \text{ x } 100$$

3.2 1D NMR Measurements

^{1}H- and ^{13}C-spectra were recorded on a *Bruker* System 600 Ascend LH, equipped with a BBO-Probe (5 mm) with z-gradient (^{1}H: 600.13 MHz, ^{13}C: 150.90 MHz,). All measurements were carried out in deuterated solvents. Resonances are reported in parts per million (ppm) relative to tetramethylsilane (TMS). The δ-scale was calibrated to the respective residual solvent signal.[1] The measured coupling constants were calculated in Hertz (Hz). The spectra were analysed using the MESTRENOVA 11.0 software. The signals were abbreviated as follows: s = singlet, bs = broad singlet, d = doublet, t = triplet, dd = doublet of doublets and m = multiplet.

3.3 Diffusion Ordered Spectroscopy (DOSY) NMR

All samples were measured in amber NMR-tubes to prevent exposure to ambient light. DOSY experiments based on ^{1}H-NMR were performed in THF-d_8 at 279.15 K on a Bruker 400 UltraShield spectrometer equipped with a Quattro Nucleus Probe (QNP) with an operating frequency of 400 MHz (^{1}H). A sequence with longitudinal eddy current delay (LED) using bipolar gradients was employed in order to compensate eddy currents. Bipolar gradient δ and a diffusion delay Δ were determined separately for each sample. Gradient strength was linearly incremented from 2% at 0.96 G to 95% at 45.7 G in 32 steps. The obtained data was processed with TopSpin 4.0.6 and Dynamics Center 2.5.3. After Fourier transformation of the 1D spectra, the signal decay along the gradients *G* was fitted to:

$$f(G) = I_0 e^{-DG^2\gamma^2\delta^2\left(\Delta - \frac{\delta}{3}\right)} \bullet 10^4$$

With the gyromagnetic ratio γ and the full signal intensity I_0.

Hydrodynamic diameters D_H were calculated from the Stokes-Einstein equation:

$$D_H = \frac{k_b T}{3\pi\eta D}$$

Where k_B is the Boltzmann constant, T the temperature and η the solvent viscosity (THF at 6 °C; extrapolated from literature: 0.572 mPa s).[2]

3.4 UV-VIS Spectroscopy

UV/vis spectra were recorded at ambient temperature on a *Shimadzu* UV-2700 spectrophotometer equipped with a CPS-100 electronic temperature control cell positioner. Samples were prepared in THF and measured in *Hellma Analytics* quartz high precision cells with a path length of 10 mm.

3.5 LC-MS Analysis

LC-MS measurements were performed on an UltiMate 3000 UHPLC system (Dionex, Sunnyvale, CA, USA) consisting of a pump (LPG 3400SZ, autosampler WPS 3000TSL) and a temperature-controlled column department (TCC 3000). Separation was performed on a C18 HPLC-column (Phenomenex Luna 5 µm, 100 Å, 250 × 2.0 mm) operating at 40 °C. A gradient of MeCN:H_2O 10:90 –> 80:20 v/v (additive 10 mmol·L^{-1} $NH_4CH_3CO_2$) at a flow rate of 0.20 mL·min^{-1} during 15 min was used as the eluting solvent. The flow was split in a 9:1 ratio, where 90 % (0.18 mL·min^{-1}) of the eluent were directed through the UV-detector (VWD 3400, Dionex, detector wavelengths 215, 254, 280, 360 nm) and 10 % (0.02 mL·min^{-1}) were infused into the electrospray source. Spectra were recorded on a LTQ Orbitrap Elite mass spectrometer (Thermo Fisher Scientific, San Jose, CA, USA) equipped with an HESI II probe. The instrument was calibrated in the m/z range 74-1822 using premixed calibration solutions (Thermo Scientific). A constant spray voltage of 3.5 kV, a dimensionless sheath gas and a dimensionless auxiliary gas flow rate of 5 and 2 were applied, respectively. The capillary temperature and was set to 300 °C, the S-lens RF level was set to 68, and the aux gas heater temperature was set to 125 °C.

3.6 Irradiation experiments with green light (λ_{max} = 525 nm):

P1 (0.05 mg mL^{-1}) and **BisTAD** (0.025 mg mL^{-1}) were dissolved in dry acetonitrile (3 mL). The reaction mixture was deoxygenized by purging with argon for 10 min. The vial was placed into a photoreactor (**Figure S19**) and irradiated with a green LED provided by Future Eden Ltd. (10 W, $\lambda_{\max}$ = 525 nm, **Figure S14)** for 20 min to obtain **SCNP1**.

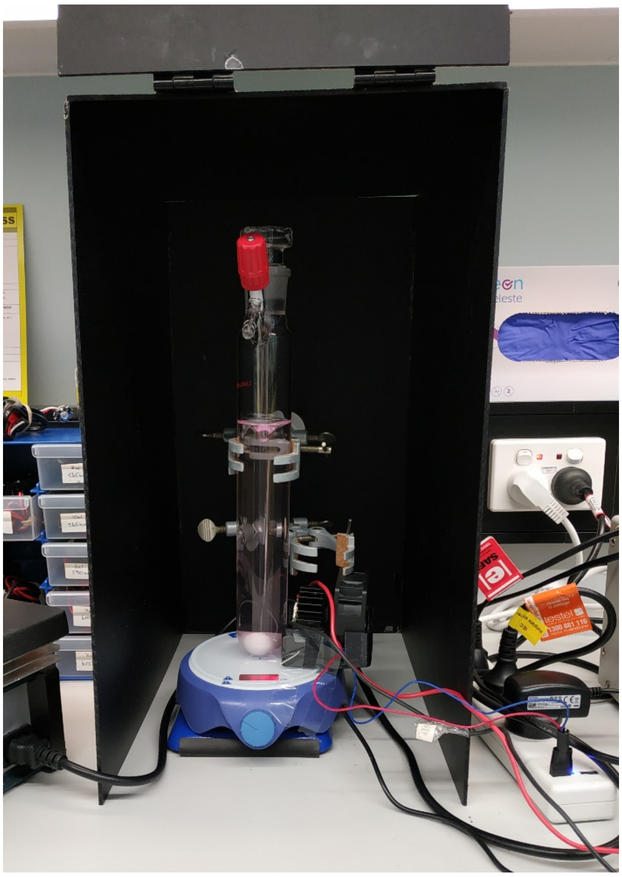

Figure S14: Irradiation setup for the photochemical experiments carried out with a green LED (10 W, λ_{max} = 525 nm, 60 mW cm^{-2}).

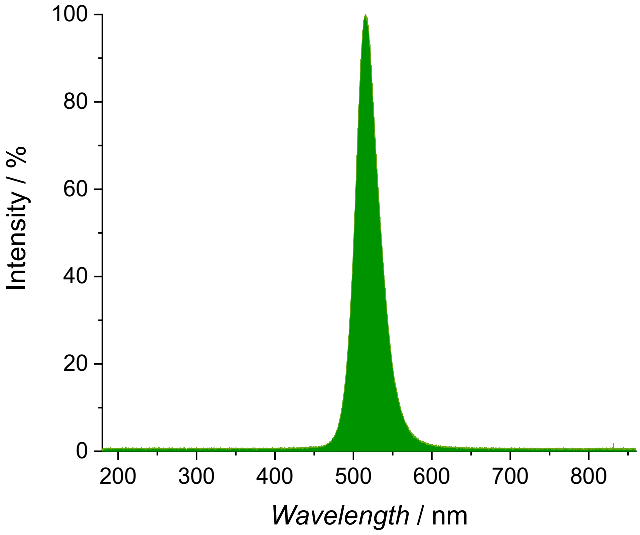

Figure S15: Emission spectrum of the green LED, centred at 525 nm.

4. Synthetic Procedures

4.1 Materials

Unless stated otherwise, all chemicals and solvents were used as received from the supplier without further purification.

2-cyanopropan-2-yl benzodithioate (Sigma-Aldrich), methyl methacrylate (Sigma Aldrich, after passing through a short plug of basic alumina), 2,2'-azobis(2-methylpropionitrile) (AIBN, Sigma-Aldrich, recrystallized), dichloromethane (DCM, Thermo Fisher Scientific, after drying and purification with SP-1 Stand Alone Solvent Purification System LC Technology Solutions Inc.), tetrahydrofuran (THF, Thermo Fisher Scientific), toluene (Thermo Fisher Scientific, after drying and purification with SP-1 Stand Alone Solvent Purification System LC Technology Solutions Inc.), hexane (Thermo Fisher Scientific), acetonitrile (Thermo Fisher Scientific, after drying and purification with SP1 Stand Alone Solvent Purification System LC Technology Solutions Inc. The solvent was subsequently again distilled using a Vigreux column, dynamically dried through molecular sieves and stored over molecular sieves), methanol (Thermo Fisher Scientific), deuterated tetrahydrofuran-d_8 (THF-d_8, Sigma-Aldrich), deuterated chloroform-d_1 ($CDCl_3$, Sigma-Aldrich), deuterated dimethylsulfoxide-d_6 (DMSO-d_6, Sigma-Aldrich), methacryloyl chloride (Sigma-Aldrich), triethylamine (dry, Sigma-Aldrich), 2-naphthoyl chloride (Combi Blocks), hexamethylene diisocyanate (Sigma-Aldrich), azodicarboxamide (Sigma-Aldrich), acetic acid (Thermo Fisher Scientific), naphthalene (Acros Organics).4-*n*-butyl-1,2,4-triazoline-3,5-dione was synthesized according to a literature procedure. [3]

4.2 Synthesis

4.2.1 Naphthalene Monomer (M1)

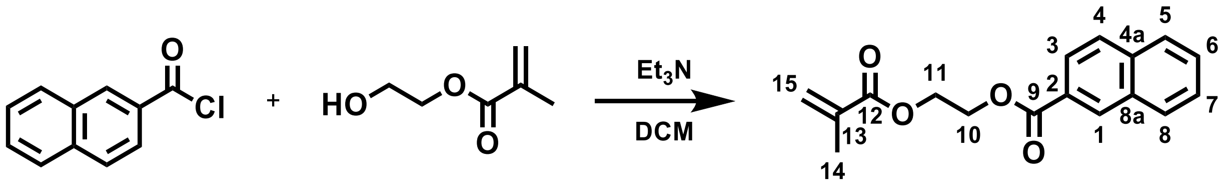

2-Naphtoyl chloride (5.00 g, 26.2 mmol, 1.0 eq.) was dissolved in dry DCM (50 mL) and cooled to 0 °C. Triethylamine (4.02 mL, 28.9 mmol, 1.1 eq.) was added to the cooled solution, followed by the dropwise addition of 2-hydroxyethyl methacrylate (3.50 mL, 28.9 mmol, 1.1 eq.). The reaction mixture was stirred overnight and was allowed to slowly warm to ambient temperature. Subsequently, 20 mL of an aqueous saturated ammonium chloride solution were added to quench the reaction and the organic phases were separated. The organic phase was dried over $MgSO_4$ and the solvent removed in vacuum. The crude product was purified with flash chromatography using a gradient (silica, ethyl acetate:cyclohexane 0:100 → 15:85). 2-(Methacryloyloxy)ethyl 2-naphthanoate was obtained as a white powder (6.81 g – 91%).

^{1}H-NMR (600 MHz, $CDCl_3$) δ (^{1}H) = 8.62 (s, 1H, CH-1), 8.06 (dd, J = 8.6, 1.7 Hz, 1H, CH-3), 7.96 (dd, J = 8.0, 1.3 Hz, 1H, CH-4), 7.91 – 7.86 (m, 2H, CH-5,8), 7.60 (ddd, J = 8.2, 6.9, 1.3 Hz, 1H,

CH-6), 7.55 (ddd, J = 8.2, 6.8, 1.3 Hz, 1H, CH-7), 6.17 (dt, J = 2.0, 1.0 Hz, 1H, CH-15), 6.00 – 5.98 (m, 1H, CH-15'), 4.66 – 4.62 (m, 2H, CH_2-10), 4.57 – 4.52 (m, 2H, CH_2-11), 1.97 (dd, J = 1.6, 1.0 Hz, 3H, CH_3-14).

^{13}C-NMR (151 MHz, $CDCl_3$) δ (^{13}C) = 167.36 (C-12), 166.65 (C-9), 136.11 (C-13), 135.77 (C-2), 132.62 (C-4a), 131.42 (C-1), 129.54 (C-4), 128.52 (C-7), 128.37 (C-5), 127.93 (C-8), 127.22 (C-8a), 126.85 (C-15), 126.28 (C-6), 125.35 (C-3), 62.91 (C-10), 62.61 (C-11), 18.44 (C-14).

LC-ESI-HRMS (m/z) Calculated for $[C_{17}H_{16}O_4H]^+$: 285.1121, measured: 285.1124.

Calculated for $[C_{17}H_{16}O_4Na]^+$: 307.0941, measured: 307.0944.

Calculated for $[C_{17}H_{16}O_4K]^+$: 323.0680, measured: 323.0682.

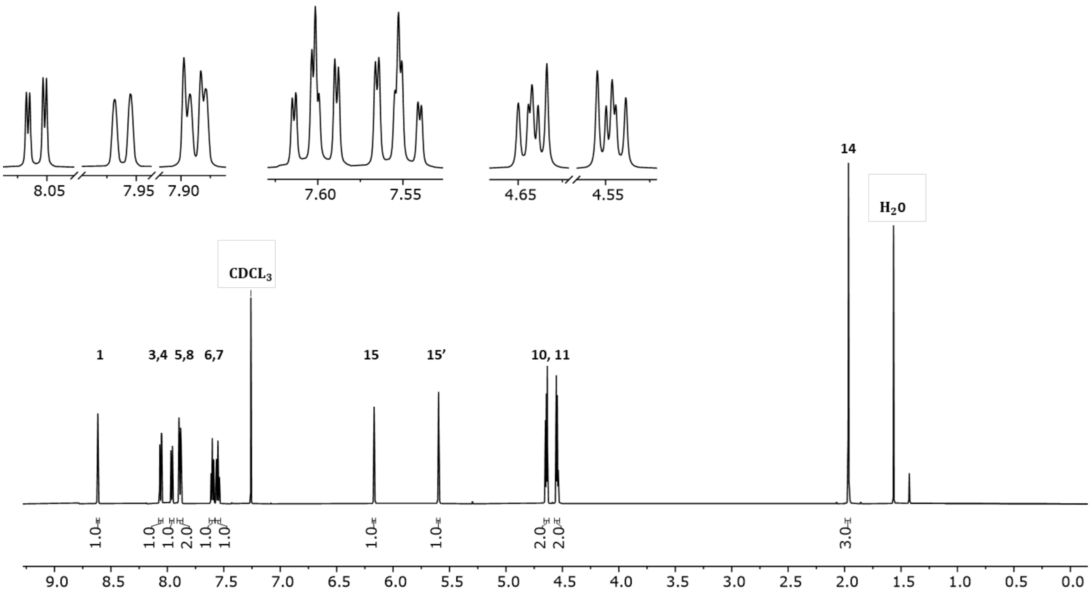

Figure S16: ^{1}H-NMR spectrum of **M1** in $CDCl_3$.

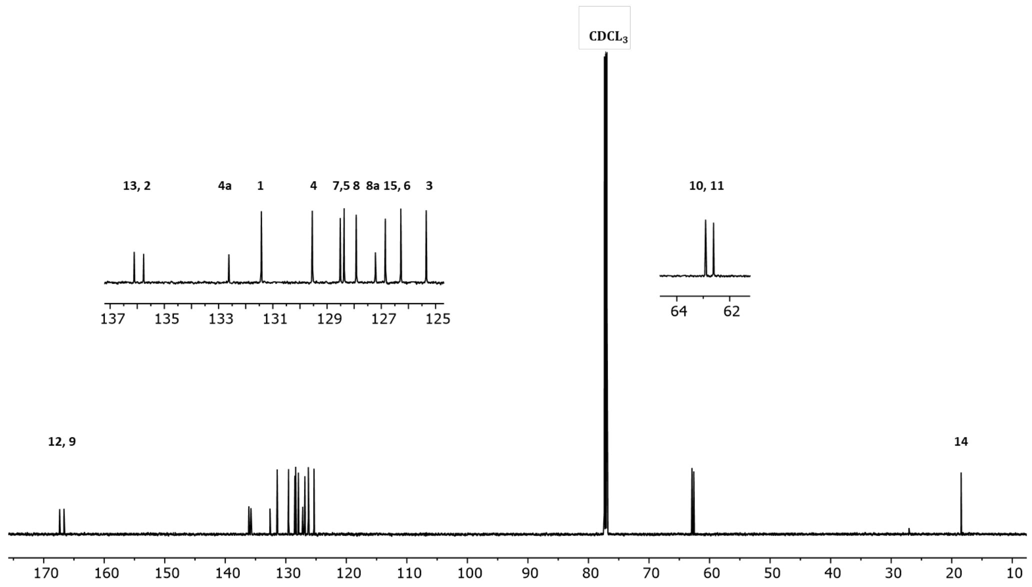

Figure S17: ^{13}C-NMR spectrum of **M1** in $CDCl_3$.

4.2.2 Synthesis of methyl 2-naphthoate (N1)

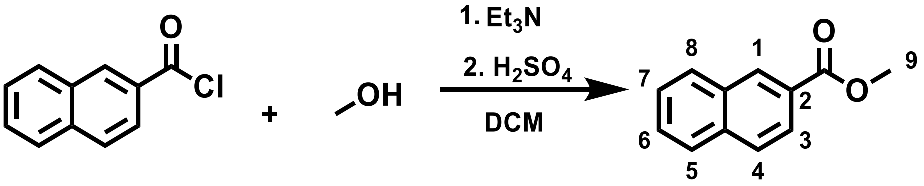

2-Naphtoyl chloride (0.50 g, 2.62 mmol, 1.0 eq.) was dissolved in dry DCM (5 mL) and cooled to 0 °C. Triethylamine (0.69 mL, 5.25 mmol, 2.0 eq.) was added to the cool solution, followed by the dropwise addition of methanol (1 mL). The reaction mixture was stirred overnight and was allowed to slowly warm to ambient temperature. Afterwards, the solution was filtered through silica to remove the base and DCM was removed in vacuum. To the dry residue, methanol (5 ml) and sulphuric acid was added, and the reaction solution was again stirred overnight, followed by the addition of DCM (20 mL) and extraction with water (2x10 mL). The organic phase was dried over $MgSO_4$ and the solvent removed in vacuum. The crude product was purified with flash chromatography using a gradient (silica, ethyl acetate:cyclohexane 0:100 → 15:85). Methyl 2-naphthoate was obtained as a white powder (0.298 g – 75%).

^{1}H-NMR (600 MHz, CD_3CN) δ (^{1}H) = 8.62 (s, 1H, CH-1), 8.07 – 8.01 (m, 2H, CH-3,4), 7.97 (dd, *J* = 8.4, 4.7 Hz, 2H, CH-5,8), 7.65 (ddd, *J* = 8.2, 6.8, 1.4 Hz, 1H, CH-6), 7.60 (ddd, *J* = 8.1, 6.8, 1.3 Hz, 1H, CH-7), 3.94 (s, 3H, CH_3-9).

LC-ESI-HRMS (m/z) Calculated for $[C_{12}H_{10}O_2H]^+$: 187.0754, measured: 187.0758.

Calculated for $[C_{12}H_{10}O_2NH_4]^+$: 204.1019, measured: 204.1026.

Calculated for $[C_{12}H_{10}O_2K]^+$: 225.0312, measured: 225.0322.

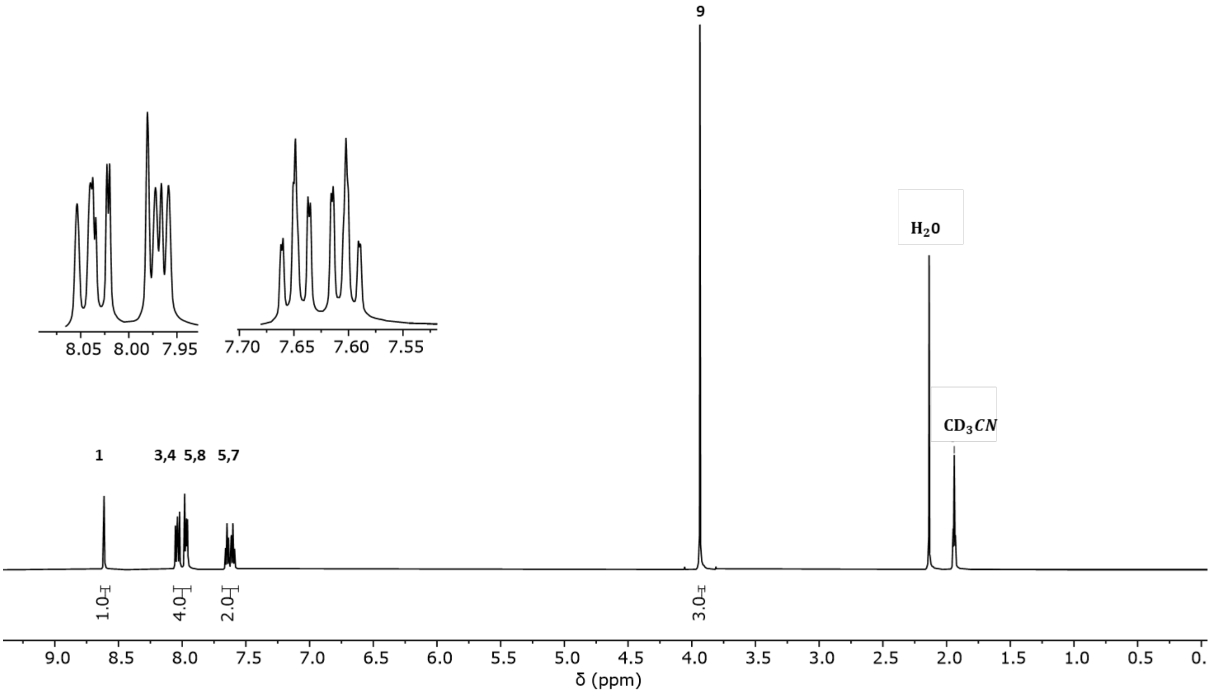

Figure S18: ^{1}H-NMR spectrum of **N1** in CD_3CN.

4.2.3 Synthesis of P1

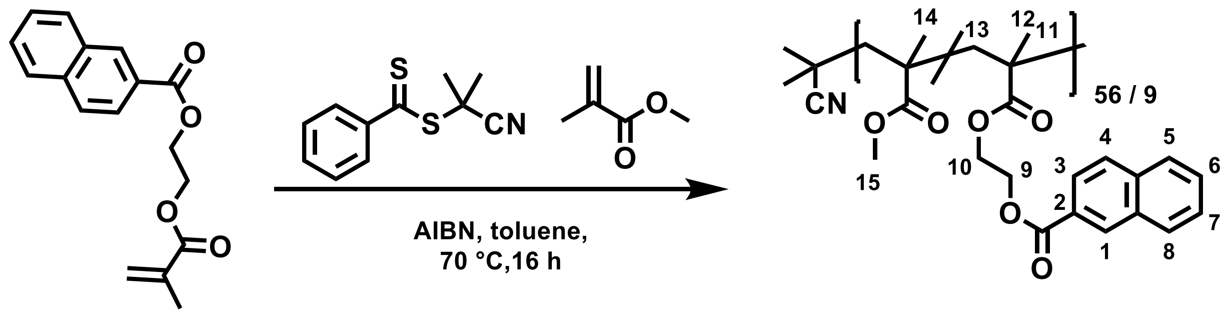

2-Cyanopropan-2-yl benzodithioate (15.00 mg, 0.067 mmol, 1.0 eq.), methyl methacrylate (542.80 g, 5.42 mmol, 80.0 eq.), **M1** (385.63 mg, 1.36 mmol, 20.0 eq.) and AIBN (2.23 mg, 0.013 mmol, 0.2 eq.) were dissolved in 1.2 mL toluene. The reaction mixture was purged with argon for 10 min and subsequently stirred at 70 °C overnight. The reaction mixture was precipitated from cold methanol, followed by dissolution in THF and precipitation in cold *n*-hexane. Afterwards, the precipitation was repeated in diethyl ether and one more time in *n*-hexane. The polymer was dissolved in a large excess of THF, the solution purged with air and

stirred overnight in an open flask to remove the RAFT end groups.[4] The residue was dissolved in THF and precipitated out of cold *n*-hexane.

Isolated yield: 480 mg (58.5 µmol).

SEC (PMMA cal.): $\overline{\overline{M}}_n$= 8,200 g mol^{-1}.

^{1}H-NMR (400 MHz, THF-d_8) δ (^{1}H) = 8.74 (bs, CH-1, 1H), 8.22 – 7.98 (m, CH-3,4,5,8, 4H), 7.66 (bs, CH-6,7, 2H), 4.63 – 4.25 (m, CH_2-9,10, 4H), 3.57 – 3.40 (m, CH_3-15, 17.8H), 2.14 – 0.72 (m, backbone).

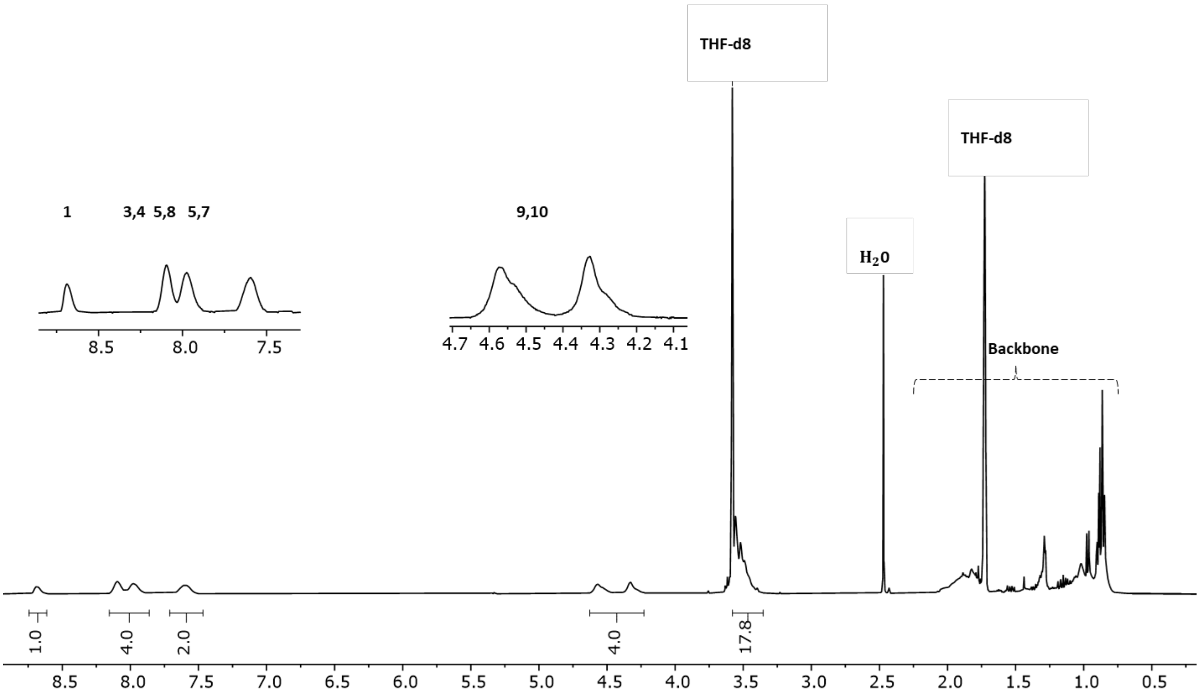

Figure S19: ^{1}H-NMR spectrum of **P1** in THF-d_8.

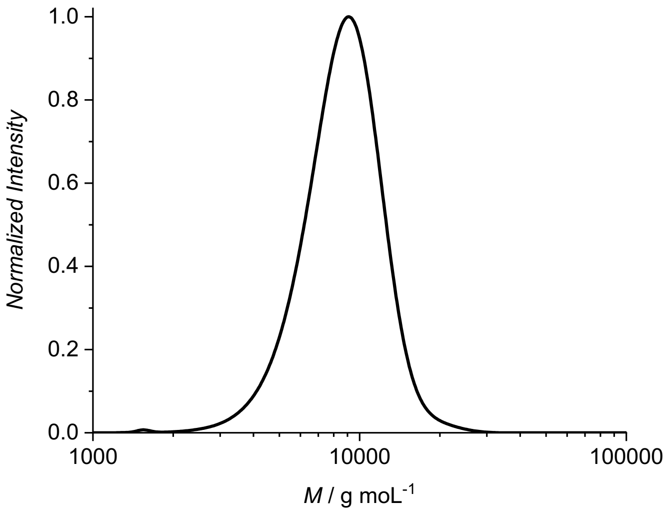

Figure S20: SEC-trace of **P1** in THF using a PMMA calibration.

4.2.4 Synthesis of P2

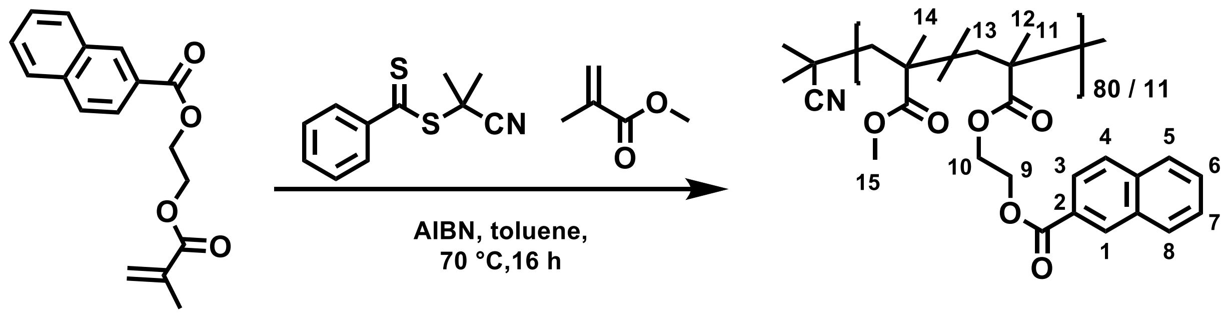

2-Cyanopropan-2-yl benzodithioate (30.00 mg, 0.135 mmol, 1.0 eq.), methyl methacrylate (1.22 g, 12.20 mmol, 90.0 eq.), M1 (385.63 mg, 1.36 mmol, 10.0 eq.) and AIBN (4.45 mg, 0.0271 mmol, 0.2 eq.) were dissolved in 1.2 mL toluene. The reaction mixture was purged with argon for 10 min and afterwards stirred at 70 °C overnight. The reaction mixture was precipitated from cold methanol, followed by resolving in THF and again precipitation in cold *n*-hexane. This was repeated in diethyl ether and again in *n*-hexane. The polymer was dissolved in a high excess of THF, the solution purged with air and stirred overnight in an open flask to remove the RAFT end groups.[4] The residue was dissolved in THF and precipitated out of cold *n*-hexane.

Isolated yield: 810 mg (72.3 µmol).

SEC (PMMA cal.): $\overline{\overline{M}}_n$ = 11,200 g mol^{-1}.

^{1}H-NMR (400 MHz, THF-d_8) δ (^{1}H) = 8.74 (bs, CH-1, 1H), 8.22 – 7.98 (m, CH-3,4,5,8, 4H), 7.66 (bs, CH-6,7, 2H), 4.63 – 4.25 (m, CH_2-9,10, 4H), 3.57 – 3.40 (m, CH_3-15, 22.9 H), 2.16 – 0.71 (m, backbone).

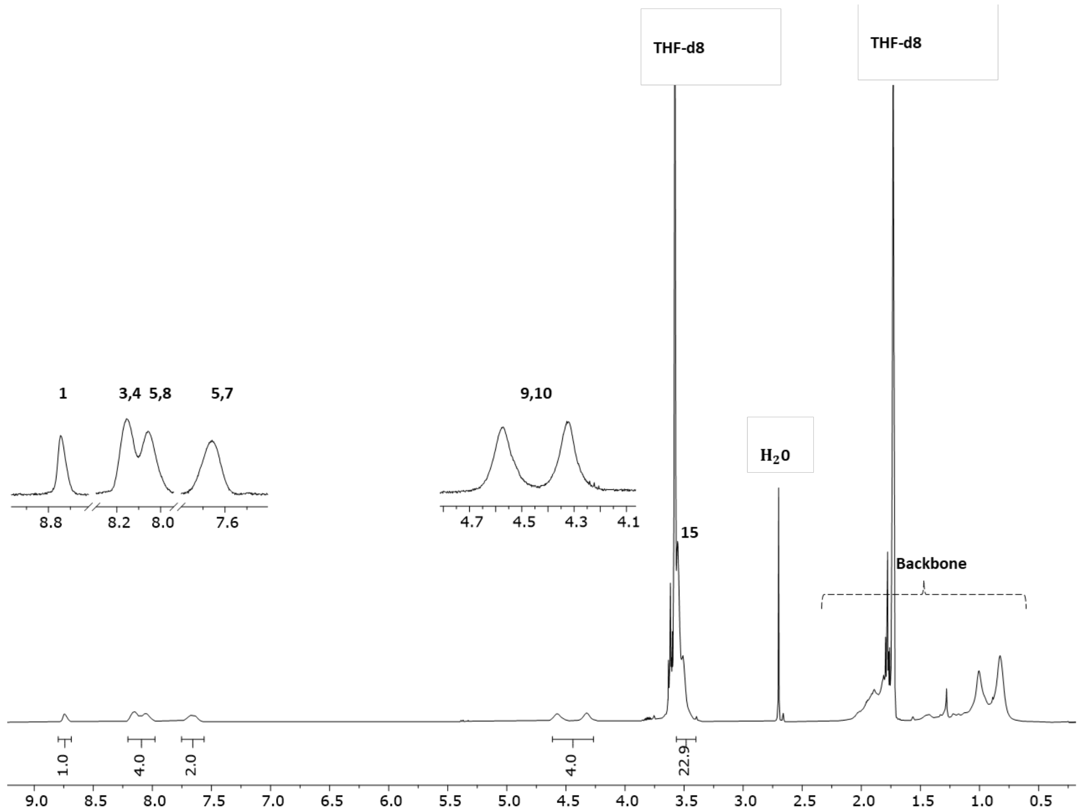

Figure S21: ^{1}H-NMR spectrum of **P2** in THF-d_8.

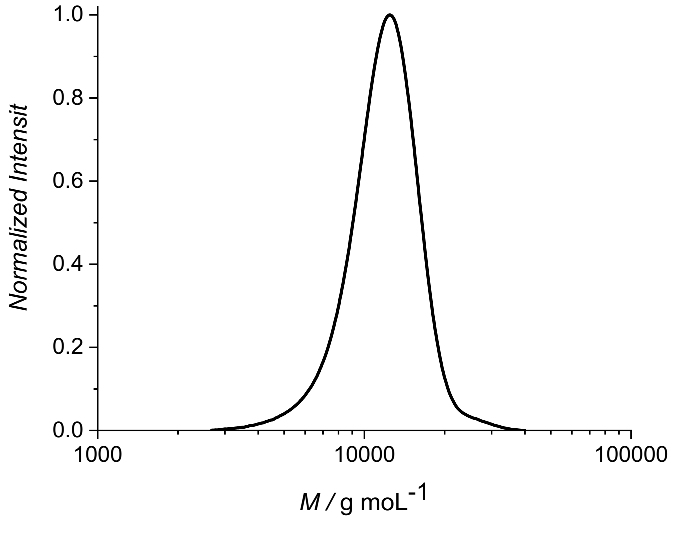

Figure S22: SEC-trace of **P2** in THF using a PMMA calibration.

4.2.5 General procedure for the folding of P1 with BisTAD

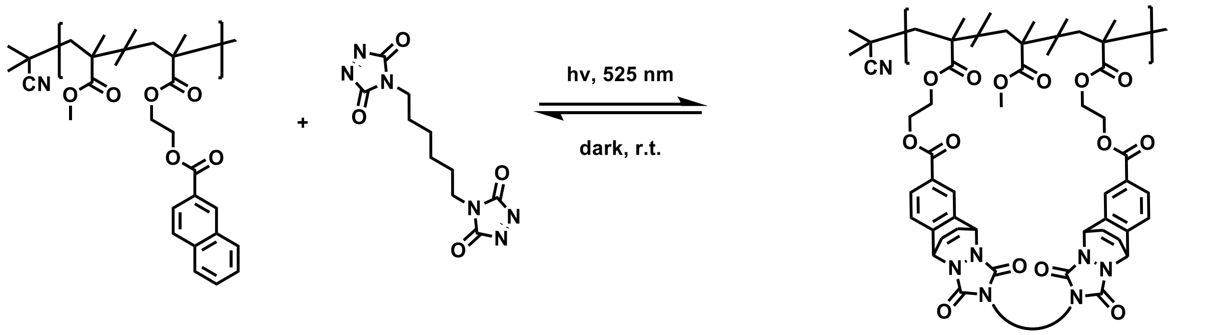

To a solution of **P1** (0.3 mg, 0.036 µmol) in 6 mL (c = 0.05 mg mL^{-1}) dry acetonitrile **BisTAD** (0.150 mg, 0.54 µmol, 3 eq.) synthesized according to a literature procedure)[3] was added. The solution was purged with argon for 10 min, followed by irradiation with a green LED for 20 min. After completion, the solution was cooled to 0 °C and the solvent removed under reduced pressure maintaining the cold temperature. The residual solid was dissolved in THF (with 0.05% Toluene as SEC standard) and injected into the SEC without further purification and delay to measure the SCNP at the maximum possible folded state.

4.2.6 General procedure for the folding of P2 with BisTAD

To a solution of **P2** (0.3 mg, 0.0268 µmol) in 6 mL (*c* = 0.05 mg mL^{-1}) dry acetonitrile BisTAD (0.125 mg, 0.44 µmol, 3 eq.) synthesized according to a literature procedure)[3] was added. The solution was purged with argon for 10 min, followed by irradiation with a green LED for 20 min. After completion, the solution was cooled to 0 °C and the solvent removed under reduced pressure maintaining the cold temperature. The residual solid was dissolved in THF (with 0.05% Toluene as SEC- standard) and directly injected into the SEC without further purification or queue time to measure the SCNP at the maximum possible folded state. For ^{1}H-NMR measurements a sephadex LH20 column using acetonitrile as eluent was performed after irradiation and cycloreversion to remove the BisTAD excess. To increase the quality of NMR measurements, Shigemi NMR-tubes for $CDCl_3$ were used.

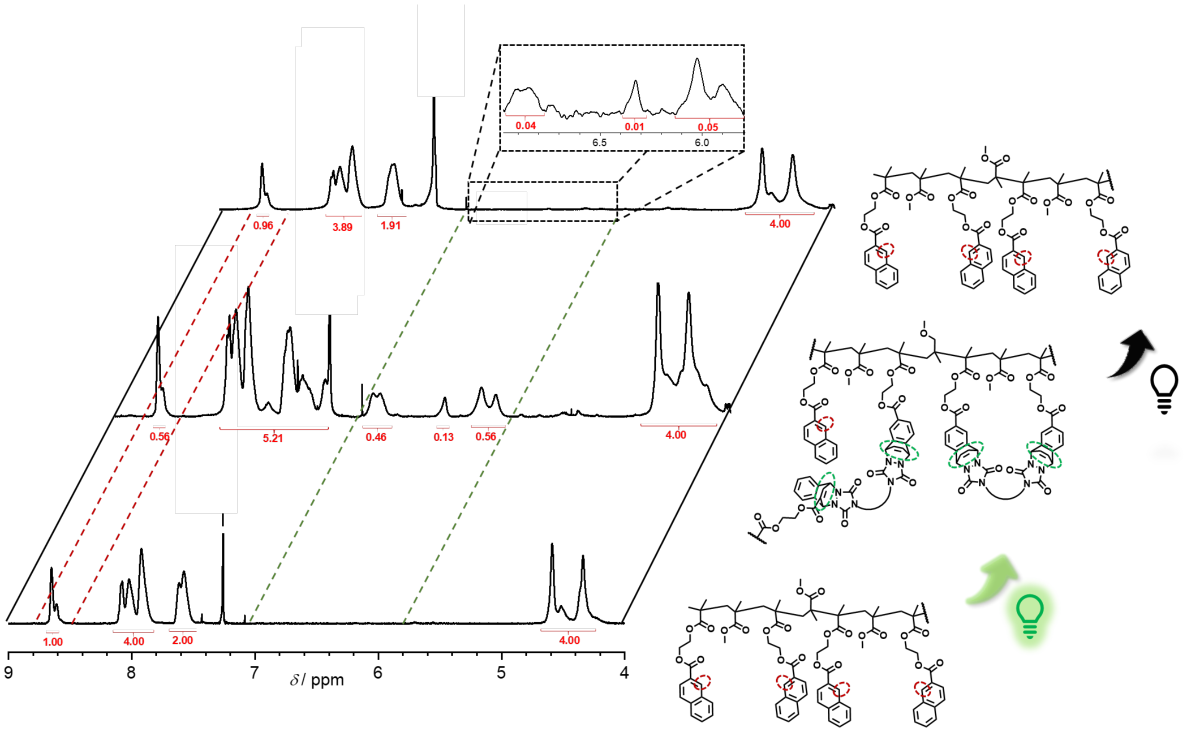

Figure S23: ^{1}H NMR spectra (in $CDCl_3$) of **P2** (bottom), **SCNP2** after irradiation with green light for 20 min (middle) and unfolding of **SCNP2** after 24h in the dark (top). The dashed red marked area displays the resolved naphthalene signal of the starting material, while the bridge protons of the cycloadducts are highlighted within the green dashed lines. Whereas the starting polymer **P2** displays the specific naphthalene pattern and no signals are visible between 6 and 7 ppm, the cycloproduct resonances become notable upon formation of the SCNP, with 45% of naphthalene units consumed (cf. red marked proton). Note that the obtained NMR of the SCNP does not represent the maximum folded state, since the sample preparation after irradiation required more than one hour, hence delaying the NMR measurement, which moreover ran for one more hour. After a total of 24 h in the dark, 96% of naphthalene side chains are present, whereas 3% of cycloproduct remain intact.

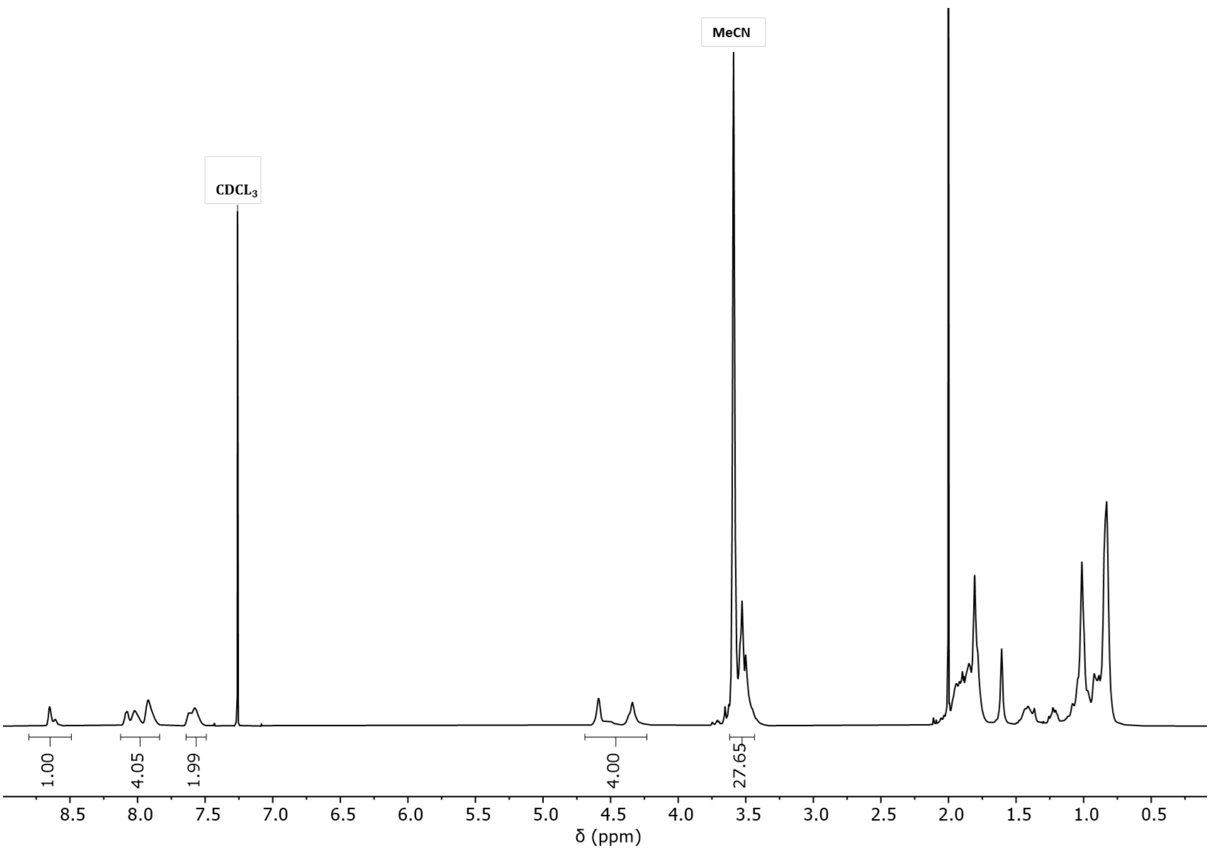

Figure S24: ^{1}H-NMR spectrum of **P2** in $CDCl_3$.

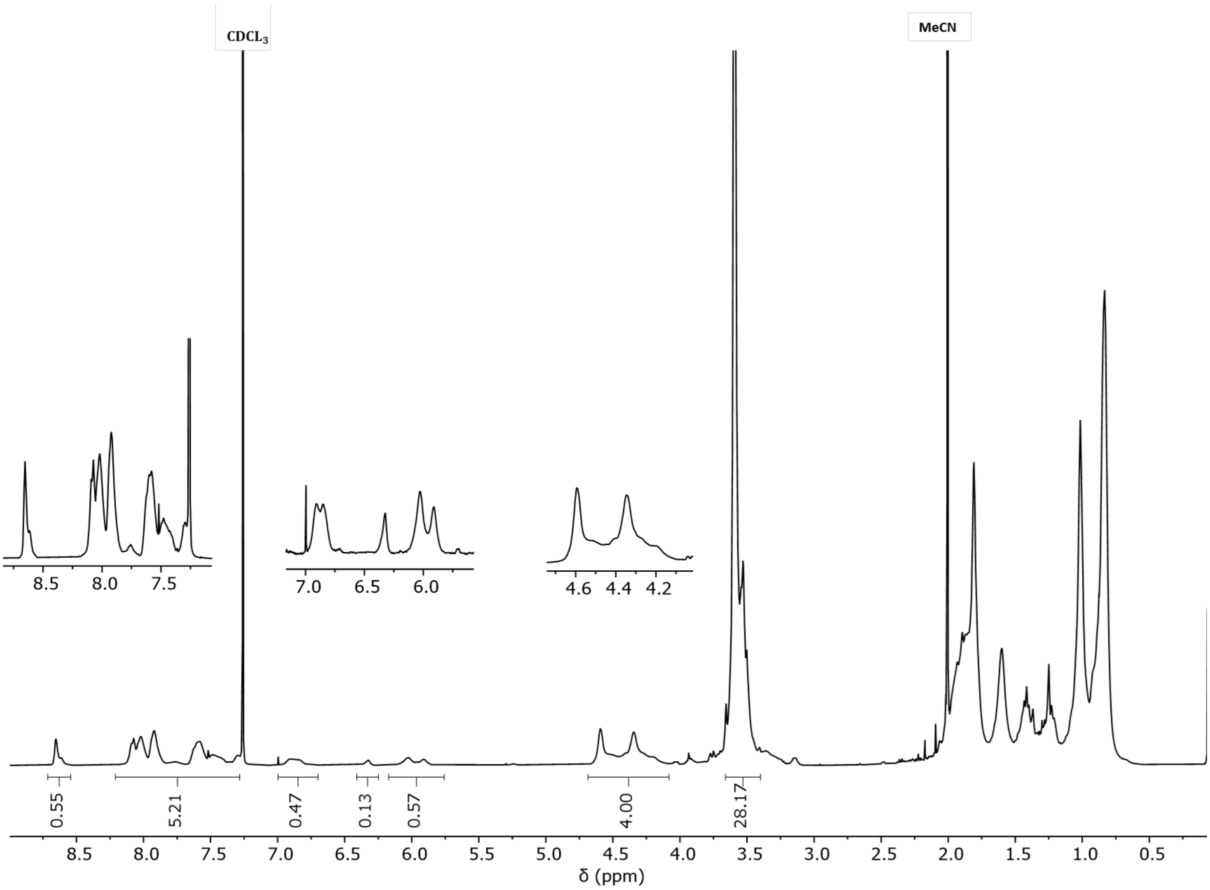

Figure S25: ^{1}H-NMR spectrum of **SCNP2** in $CDCl_3$.

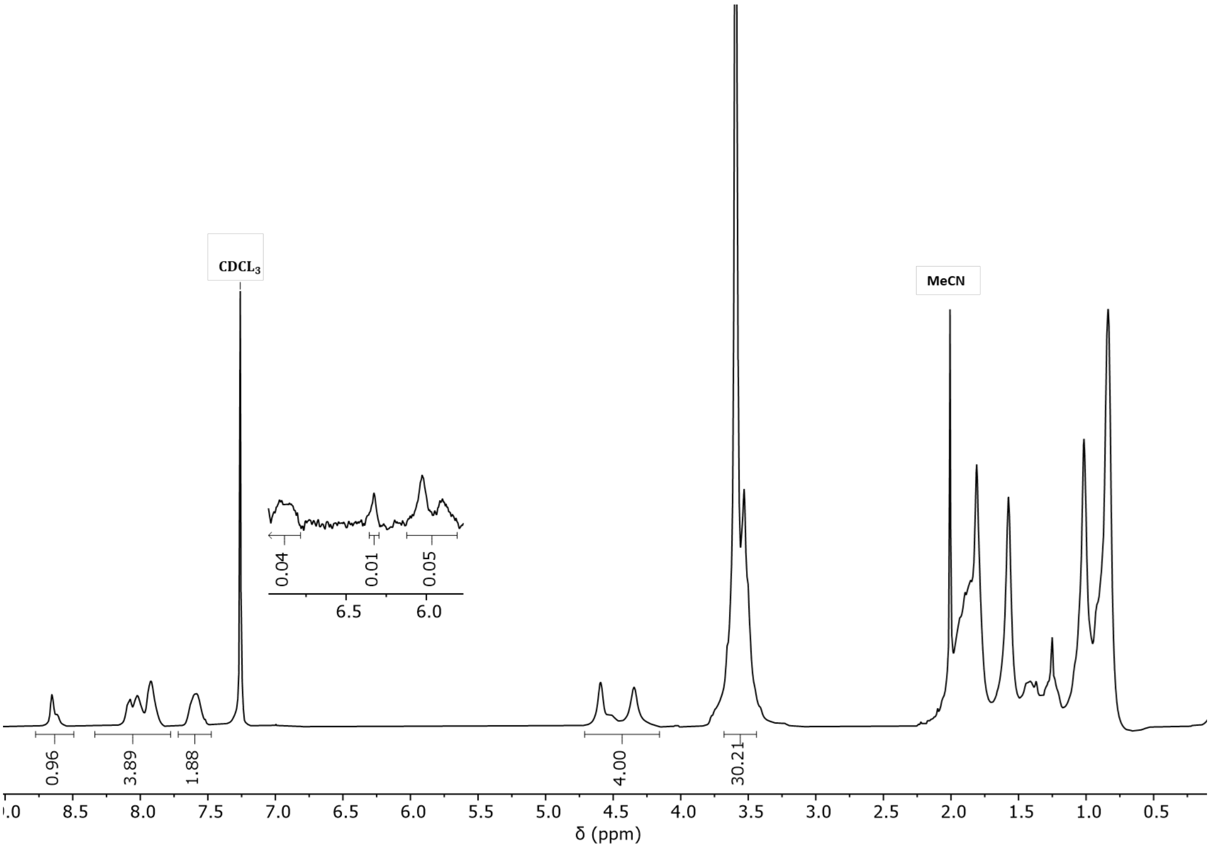

Figure S26: ^{1}H-NMR spectrum of **SCNP2** after 24h in the dark in $CDCl_3$.

4.2.7 Procedure for the light stabilization of naphthalene N1

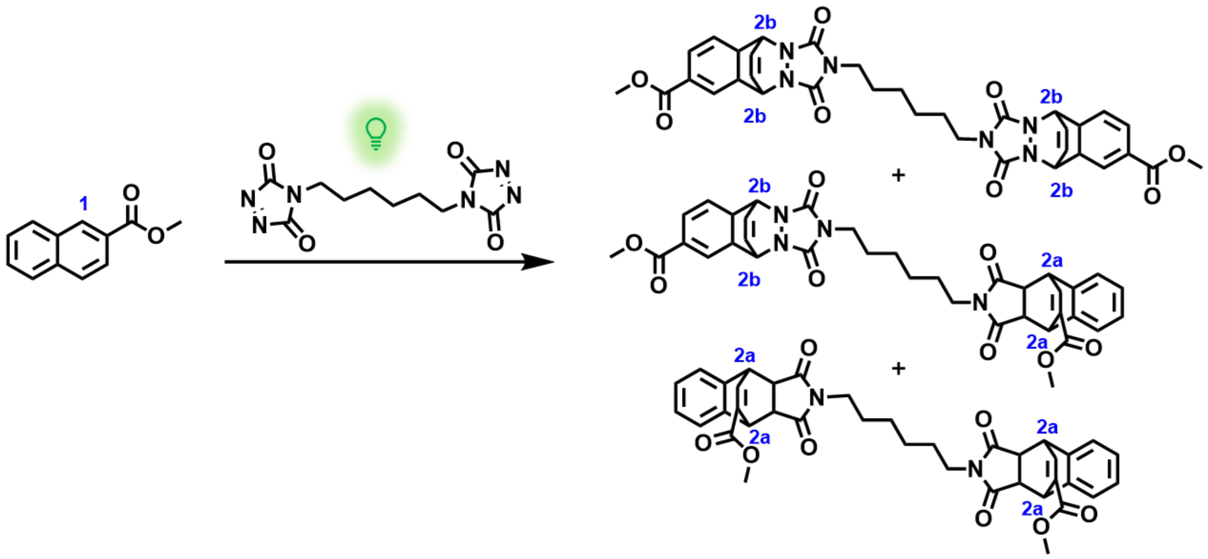

Naphthalene **N1** (2.5 mg, 0.13 mmol, 1.0 eq) and **BisTAD** (1.5, 0.0054 mmol, 0.4 eq.) were dissolved in deuterated acetonitrile (0.7 mL, dried one week over molecular sieves) and the solution transferred into an NMR-tube. Argon was purged through the reaction solution for deoxygenation and the NMR tube was closed. Under exclusion from light, ^{1}H-NMR was measured followed by irradiation for 40 min (2 cm distance, 10 W, λ_{max} = 525 nm). The NMR-tube was removed from the light source and ^{1}H-NMR was measured immediately. After completed measurement, the samples were put back into the setup and irradiation was proceeded. Using the same procedure, ^{1}H-NMR measurements were undertaken at 16 h, 24 h respectively. The percentage of dimer was calculated from the integration of proton 1 of the staring material **N1** relative to the bridge protons 2a and 2b of the Diels-Alder cycloadducts. The overall product mixture can contain three different regio-isomers, as displayed in the reaction scheme. An exemplary ^{1}H-NMR spectrum, obtained after the first irradiation, is shown here below.

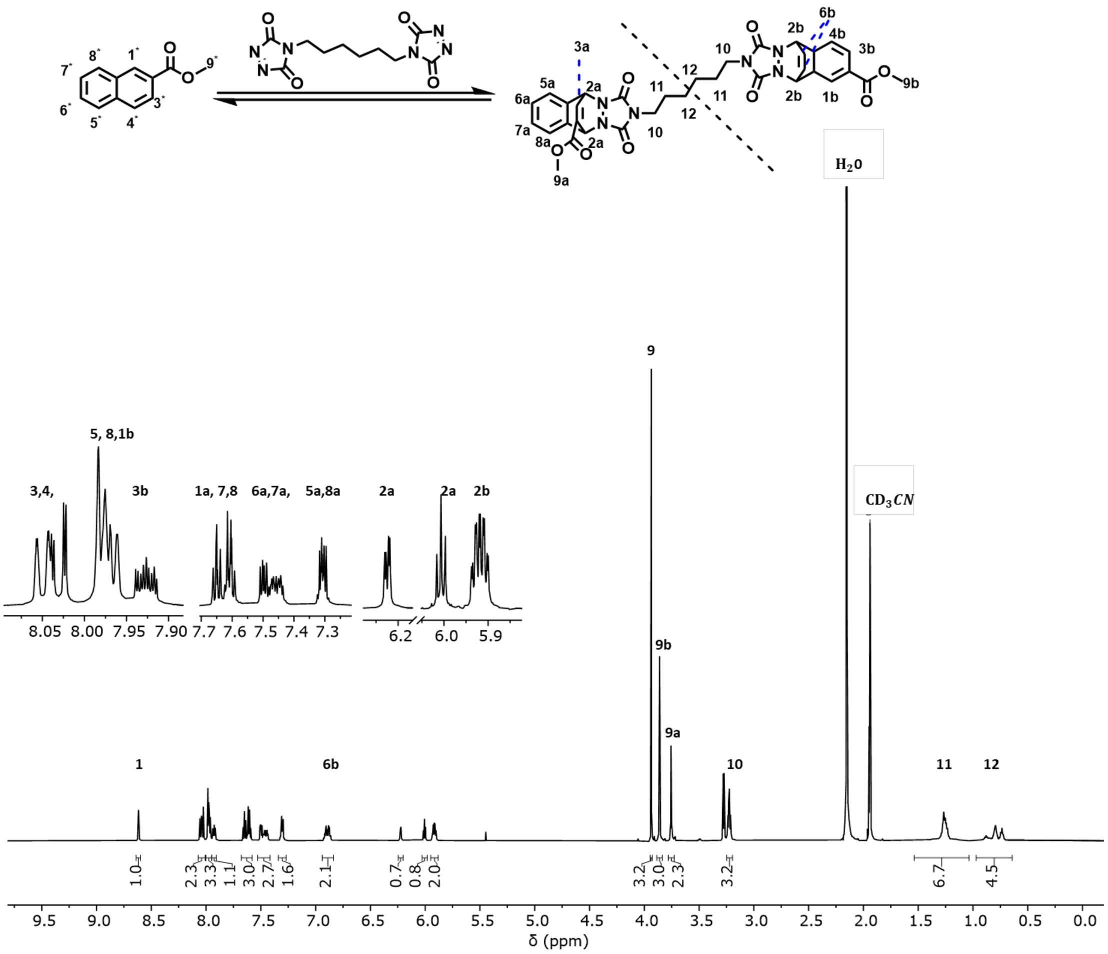

Figure S27: ^{1}H-NMR spectrum of the irradiation products of **N1** and **BisTAD** in CD_3CN.

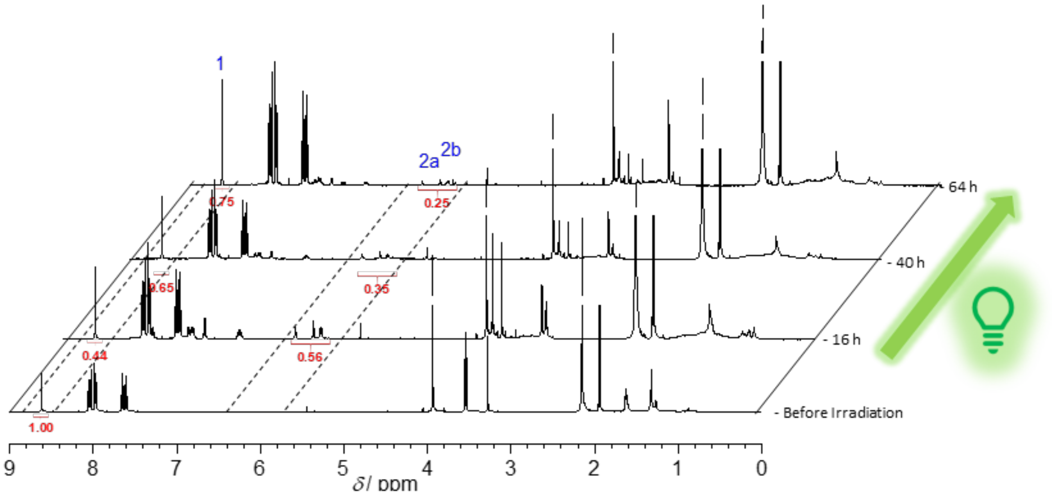

Figure S28: Additional ^{1}H-NMR spectrum to investigate the light-stabilization of the irradiation products of **N1** and **BisTAD** in CD_3CN.

4.2.8 Procedure for ^{1}H-NMR measurements of reversible cycloaddition/cycloreversion of naphthalene N1 with BisTAD

4.2.8.1 Cycloaddition/cycloreversion of naphthalene **N1** with **BisTAD**

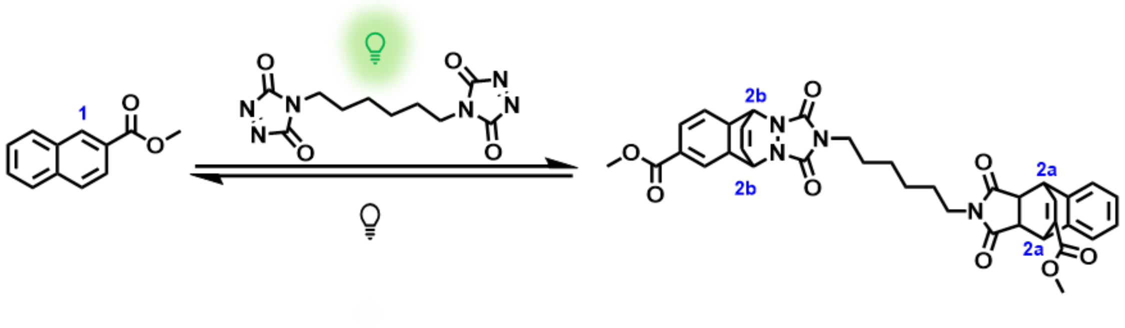

Naphthalene **N1** (2.5 mg, 0.013 mmol, 1.0 eq) and **BisTAD** (1.5, 5.35 mmol, 0.4 eq.) were dissolved in deuterated acetonitrile (0.7 mL, dried one week over molecular sieves) and the solution transferred to an NMR-tube. Argon was purged through the reaction solution for deoxygenation and the NMR tube was sealed. Under exclusion from light, ^{1}H-NMR was measured followed by irradiation for 40 min (2 cm distance, 10 W, λ_{max} = 525 nm), leading to a loss of color (Figure S25). The NMR-tube was removed, and ^{1}H-NMR was measured immediately. After complete measurement, the samples were covered with aluminium foil and stored at ambient temperature for 24 h, during which the color was regained (Figure S25).

^{1}H-NMR was measured afterwards, and the samples were irradiated again for 40 min, followed by the 24 h of darkness. After three consecutive cycles, the sample was stored for 4 additional days in the dark to investigate the final reaction outcome (8% Dimer, see figure S26). The percentage of dimer to naphthalene **N1** was calculated by comparing proton 1 of staring material **N1** to the bridge protons 2a and 2b of the Diels-Alder adducts.

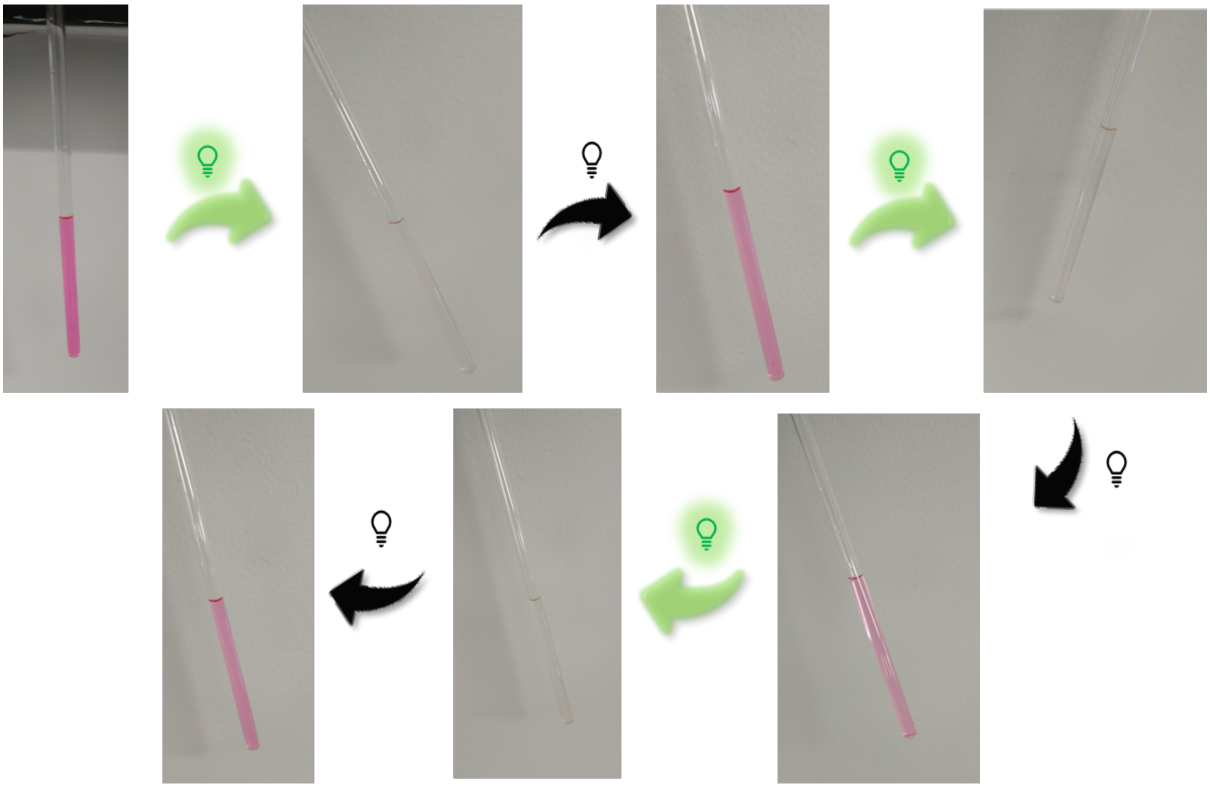

Figure S29: Pictures of the NMR-tube during the reversible cycloaddition/reversion cycles of **BisTAD** with naphthalene **N1**, displayed in Figure 5 main paper. All pink samples display the presence of free TAD-moieties, whereas after irradiation a colorless solution is obtained.

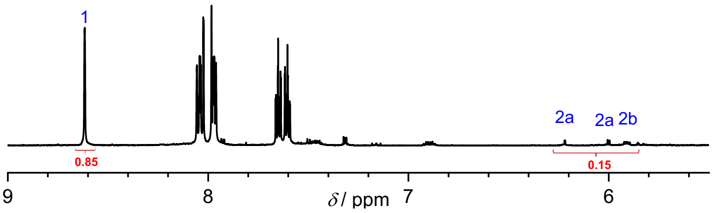

Figure S30: Additional ^{1}H-NMR spectrum of an **N1** and **BisTAD** mixture obtained 4 days after the last reversion cycle, measured in CD_3CN, showing around 8% of cycloadduct still remaining.

4.2.8.2 Cycloaddition/cycloreversion of naphthalene **N1** with **4-*n*-butyl-TAD**

A solution of 4-*n*-butyl-1,2,4-triazoline-3,5-dione (3.53 mg, 0.023 mmol, 1.0 eq.) and methyl 2-naphthoate (**N1**, 5.03 mg, 0.027 mmol, 1.2 eq.) in 1.5 mL deuterated acetone-d_6 (15 mM) was divided over two NMR-tubes.

A first NMR tube was subjected to green LED irradiation (λ = 515 – 525 nm, 3 x 3 W) for 45 min, at which complete photobleaching was observed. Immediately after irradiation, the resulting clear and colorless solution was submitted for ^{1}H NMR analysis (see Figures S31a for the corresponding spectrum). Two regioisomers C_A and C_B (C_A:C_B = 42:58) were identified, i.e. the cycloadduct formed upon addition of TAD onto the substituted and non-substituted ring of **N1**, respectively.

^{1}H-NMR (400 MHz, Me_2CO-d_6) δ (^{1}H) for C_A = 7.73 (dd, 1H, Ar-CH-C*H*=C, *J* = 6.1, 1.8 Hz), 7.49-7.59 (m, 2H, Ar*H*), 7.34 (m, 2H, Ar*H*), 6.26 (d, 1H, Ar-C*H*-N, *J* = 1.5 Hz), 6.12 (d, 1H, Ar-C*H*-N, *J* = 6.4 Hz), 3.79 (s, 3H, OCH_3), 3.32 (t, 2H, N-CH_2, *J* = 6.8 Hz), 1.39 (m, 2H, N-CH_2-CH_2), 0.98 (m, 2H, CH_3-CH_2), 0.78 (t, 3H, CH_3, *J* = 7.2 Hz). Some resolved resonances δ (^{1}H) for C_B = 8.06 (m, 1H, Ar*H*), 7.96 (m, 1H, Ar*H*), 7.62 (m, 1H, Ar*H*), 6.97 (m, 2H, Ar-CH-C*H*=CH), 6.04 (dd, 1H, Ar-C*H*-N, *J* = 5.3, 1.9 Hz), 6.01 (dd, 1H, Ar-C*H*-N, *J* = 5.2, 1.6 Hz), 3.89 (s, 3H, OCH_3).

The recovered samples was then kept in the dark (wrapped in aluminium foil) and stored at 25 °C for 60 h, after which a ^{1}H NMR spectrum was recorded. Integration of the well-resolved signals in the ^{1}H NMR spectra allowed to determine the concentration of remaining TAD/naphthalene cycloadduct (see Figure 31b).

The second NMR tube was kept in the dark at 25 °C (wrapped in aluminium foil) throughout the cycloreversion study. It hence served as a non-irradiated reference to assess the cleanliness of the cycloreversion process (see Figure S31c).

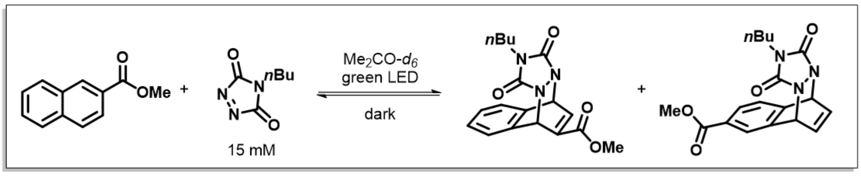

a) after 45 min green light:

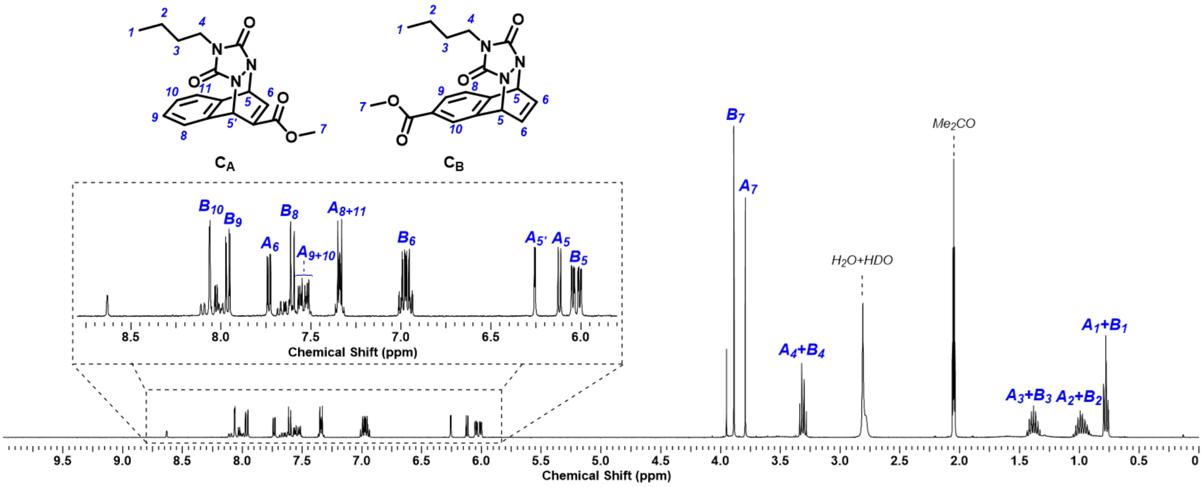

b) after 45 min green light, followed by 60 h in the dark:

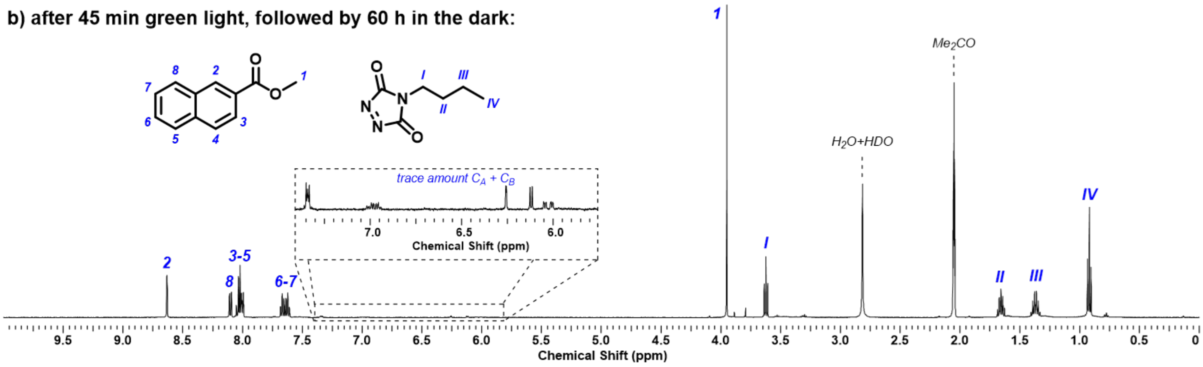

c) non-irradiated reference sample, kept in the dark for 60 h:

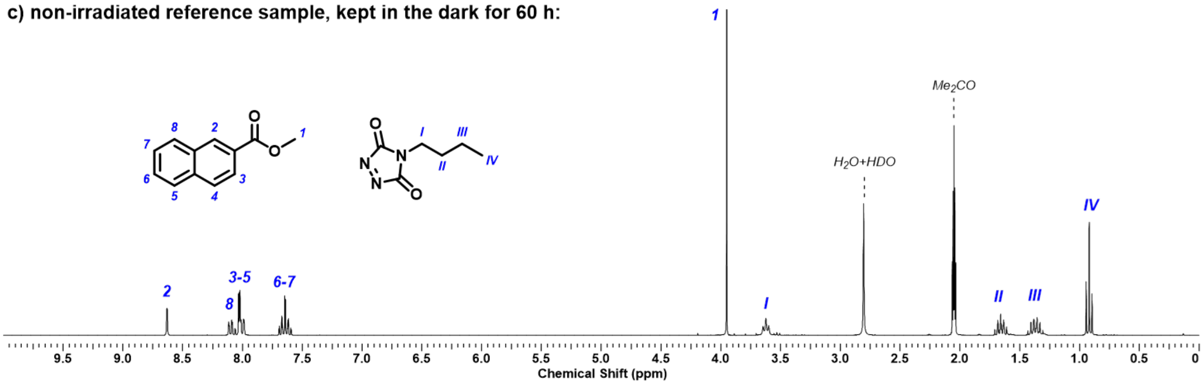

Figure S31: ^{1}H-NMR analysis of the cycloadduct formation and -reversion of the methyl 2-naphthoate (**N1**)/**4-*n*-butyl-TAD** system. a) ^{1}H NMR spectrum (recorded in Me_2CO-d_6) of the TAD/naphthalene cycloadducts, formed upon 45-min green light irradiation (λ = 515 – 525 nm, 3 x 3 W LEDs) of **4-*n*-butyl-TAD** (15 mM, Me_2CO-d_6) in the presence of methyl 2-naphthoate (1.2 eq.). Both regioisomers C_A and C_B, i.e. the cycloadduct formed onto the substituted and non-substituted ring, respectively as well as the complete consumption of TAD can be detected. b) ^{1}H NMR spectrum (in Me_2CO-d_6) of the irradiated TAD/naphthalene mixture upon standing in the dark for 60 h, indicating a clean cycloreversion process with less than 7 % cycloadduct remaining. c) ^{1}H NMR spectrum (in Me_2CO-d_6) of a **N1**/**4-*n*-butyl-TAD** reference sample, kept in the dark for 60 h without being subjected to green light.

4.2.9 Procedure for cycloaddition/cycloreversion exchange experiment under continuous irradiation

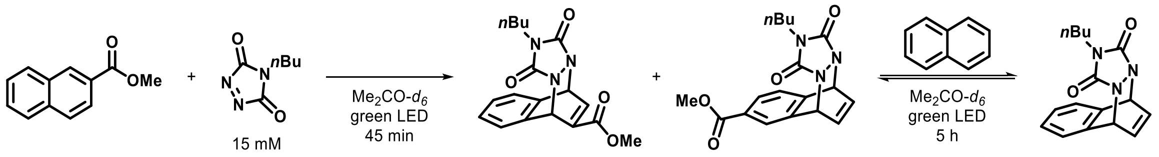

A purple solution of 4-*n*-butyl-1,2,4-triazoline-3,5-dione (3.53 mg, 0.023 mmol, 1.0 eq.) and methyl 2-naphthoate (**N1**, 5.03 mg, 0.027 mmol, 1.2 eq.) in 1.5 mL deuterated acetone-d_6 (15 mM) was subjected to green LED irradiation (λ = 515 – 525 nm, 3 x 3 W) for 45 min, at which complete photobleaching was observed. Next, a solution of plain naphthalene (3.46 mg, 0.027 mmol, 1.2 eq.) in 0.2 mL deuterated acetone-d_6 was added to the colorless cycloadduct solution.

The resulting mixture was submitted for ^{1}H NMR analysis (see Figures S32a for the corresponding spectrum) and divided over two equal samples, whereby a first was immediately re-subjected to green LED light for an additional 5 hours, while a second one was placed in the dark (wrapped in aluminium foil) hence serving as a reference sample. Whereas the former sample remained colorless throughout the irradiation process, the latter slowly regained a pink color over time.

Both samples were submitted for ^{1}H NMR analysis (see Figures S31b-c) in order to identify whether an exchange of cycloadducts occurred under continuous irradiation, and thus whether the initial TAD/naphthalene cycloadduct remained their dynamic behaviour.

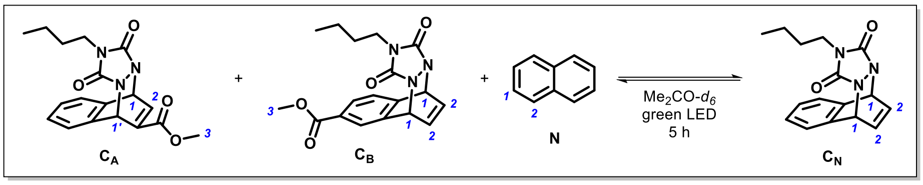

a) after 45 min green light, followed by naphthalene addition

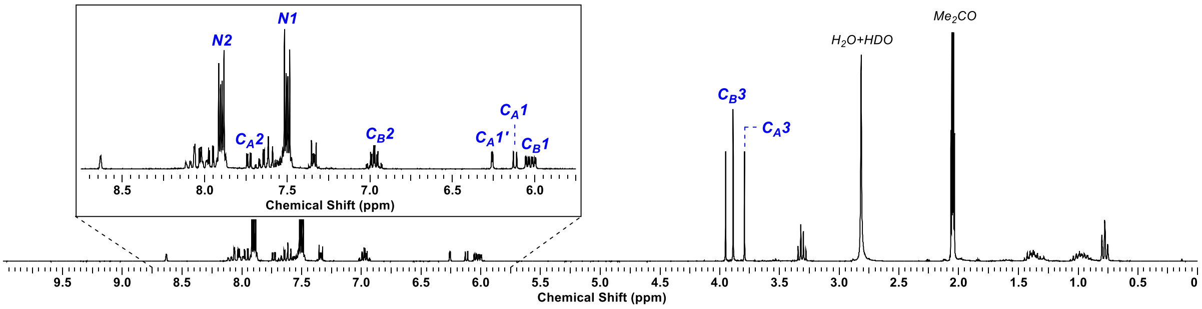

b) resulting mixture kept in the dark for 5 h:

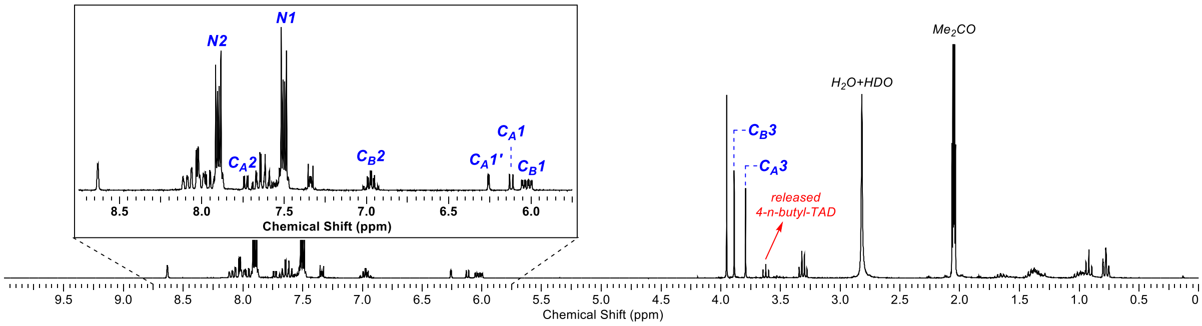

c) resulting mixture after 5 h additional green light

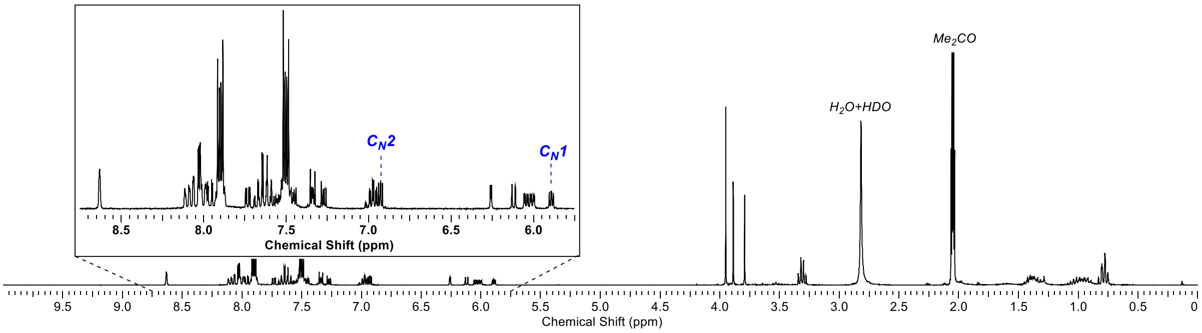

d) reference spectrum of 4-n-butyl-TAD + naphthalene after 45 min green light

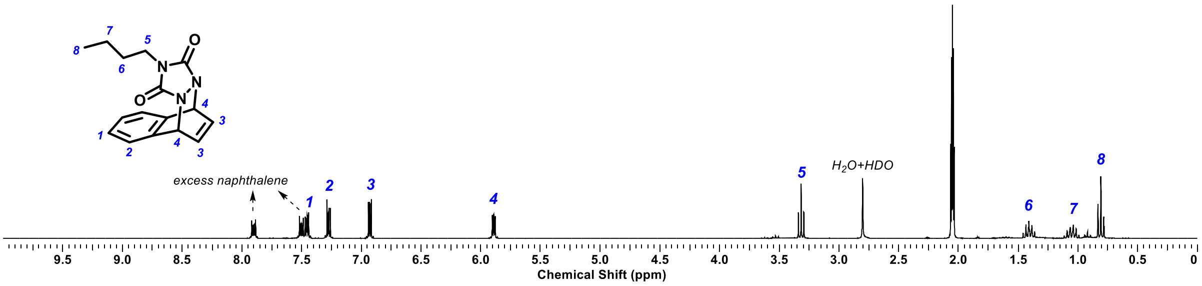

Figure S32: ^{1}H NMR analysis of the exchange experiment of the methyl 2-naphthoate (**N1**)/**4-*n*-butyl-TAD** system with plain naphthalene under continuous irradiation. a) ^{1}H NMR spectrum (recorded in Me_2CO-d_6) of the initial cycloadduct mixture, formed upon 45-min green light irradiation (λ = 515 – 525 nm, 3 x 3 W LEDs) of **4-*n*-butyl-TAD** (15 mM, Me_2CO-d_6) in the presence of methyl 2-naphthoate (1.2 eq.), following the addition of 1.2 eq. plain naphthalene. b) Whereas upon standing in the dark for 5 h, only the cycloreversion of the **N1**/**4-*n*-butyl-TAD** system is observed, c) continued irradiation with green light results in the formation of a new TAD-cycloadduct with plain naphthalene. d) Comparison with a reference spectrum of the latter cycloadduct indeed evidenced the initially formed cycloadduct to retain their dynamic behavior, allowing for an exchange reaction to take place.

4.2.10 General procedure for light-fueled stabilization of P2.

After SCNP2 was obtained (see general procedure for folding 4.2.6) one sample was taken out under argon gas flow for SEC measurement and the residual solution continuously irradiated. This was repeated at 8 h, 48 h and 72 h.

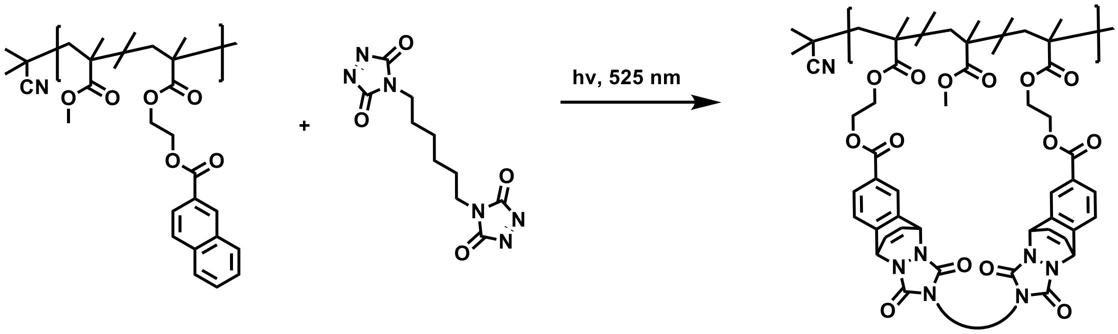

4.2.11 Procedure for UV/Vis measurements of reversible cycloaddition/cycloreversion of naphthalene N1

4.2.11.1 Relatively high concentrations (NMR-concentrations)

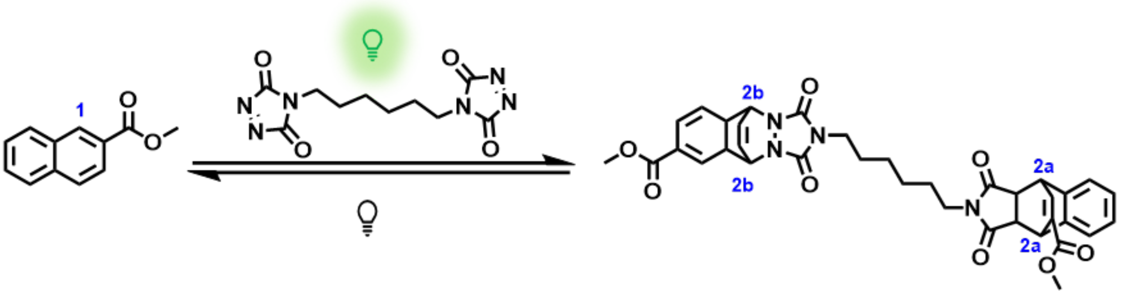

Naphthalene **N1** (5.5 mg, 0.0030 mmol, 1.0 eq.) and **BisTAD** (3.5, 0.013 mmol, 0.4 eq.) were added to a 10 mL Schlenk flask and dissolved in dry acetonitrile (1.5 mL). Argon was bubbled through the reaction solution for deoxygenation and the sample was irradiated for 40 min (2 cm distance, 10 W LED, $\lambda_{\max}$ = 525 nm). Under argon flow, 0.1 mL of the now colorless reaction solution was taken out using an Eppendorf pipette and added to a UV/Vis cuvette. The flask was then covered in aluminum foil and stored for 24 h at ambient temperature. 2 mL acetonitrile was added to the cuvette and UV/Vis absorbance was measured over a period of 24 h. After 24 the retrieved pink reaction solution in the Schlenk flask was irradiated again for 40 min and submitted for UV/Vis measurement, following the described procedure. The procedure was repeated for three consecutive cycles.

4.2.11.2 Diluted concentrations

To investigate the influence of diluted concentration on the reversibility, which is critical for the typically used highly dilute concentrations of SCNP folding, the previously described UV/Vis experiments were repeated at lower concentrations. Therefore, naphthalene **N1** (0.40 mg, 0.0022 mmol, 1.0 eq) and **BisTAD** (0.24, 0.0008 mmol, 0.4 eq.) were dissolved in dry acetonitrile (2 mL) and transferred into a cuvette. The solution was deoxygenated, and UV/Vis was measured, followed by irradiation for 40 min. After irradiation, UV/Vis was measured for 24 h.

4.2.12 General procedure for chemical SCNP stabilization upon reduction

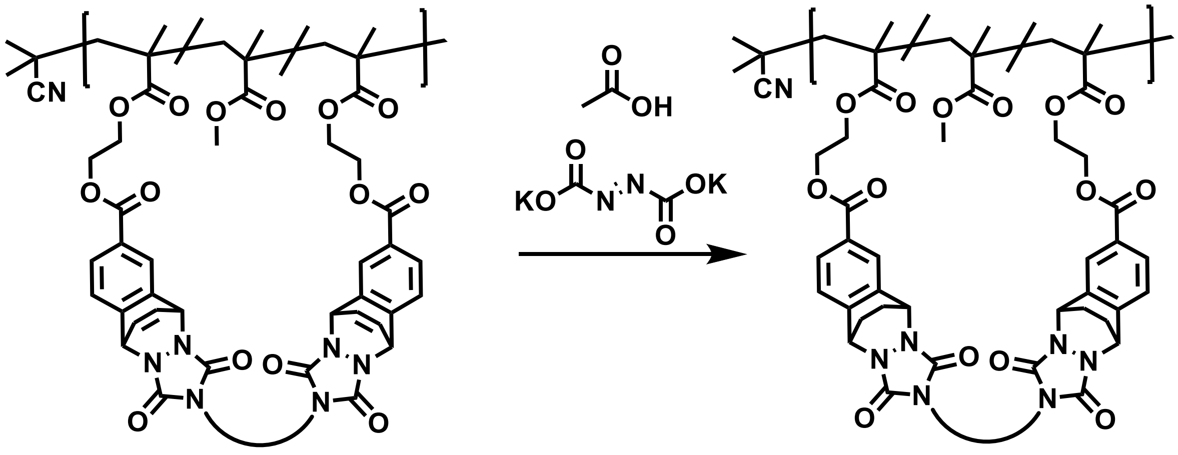

The procedure was carried out following a literature procedure.[5]

After the folding process (see general procedure for folding 4.2.5), the dry polymer was dissolved in a small amount of ethyl acetate and cooled to 0 °C. To this, acetic acid and potassium azodicarboxylate (PAD, synthesized according to literature)[6] were added to the solution. The reaction mixture was stirred for 2 h and then allowed to warm up to room temperature for SEC analysis.

4.2.13 General procedure for light-fueled stabilization of P1.

The obtained SCNP (see general procedure for folding 4.2.5) was continuously irradiated and samples were taken under inert gas flow.

4.2.14 General procedure for the kinetic trapping of SCNPs below ambient temperature

The SCNP obtained after irradiation (see general procedure for folding 4.2.5) was immediately covered in aluminium foil to prevent it from being exposed to light and cooled in an ice bath. Subsequently, the sample was transferred into the fridge or freezer, respectively. For sample analysis, the solvent was removed under reduced pressure, maintaining temperatures below ambient temperature, and submitted to SEC measurement.

5. References

1. G. R. Fulmer, A. J. M. Miller, N. H. Sherden, H. E. Gottlieb, A. Nudelman, B. M. Stoltz, J. E. Bercaw and K. I. Goldberg, *Organometallics*, 2010, **29**, 2176-2179.
2. D. R. Lide, Ed. , in CRC Handb. Chem. Phys., CRC Press, Boca Raton, Florida, 2005.
3. H. A. Houck, E. Blasco, F. E. Du Prez and C. Barner-Kowollik, *J. Am. Chem. Soc.*, 2019, **141**, 12329-12337.
4. H. Frisch, F. R. Bloesser and C. Barner-Kowollik, *Angew. Chem. Int. Ed.*, 2019, **58**, 3604-3609.
5. M. Okumura, S. M. Nakamata Huynh, J. Pospech and D. Sarlah, *Angew. Chem. Int. Ed.*, 2016, **55**, 15910-15914.
6. J. T. Groves and K. W. Ma, *J. Am. Chem. Soc.*, 1977, **99**, 4076-4082.

6. Primary DOSY Data – Appendix

All DOSY spectra were measured at 6°C to maintain the present state.

Measured samples and abbreviations employed in the DOSY files:

SAMPLE	DOSY FILE
P2	DAK_042_C
SCNP2	DAK_097
SCNP2 after 24h	DAK_097_24h

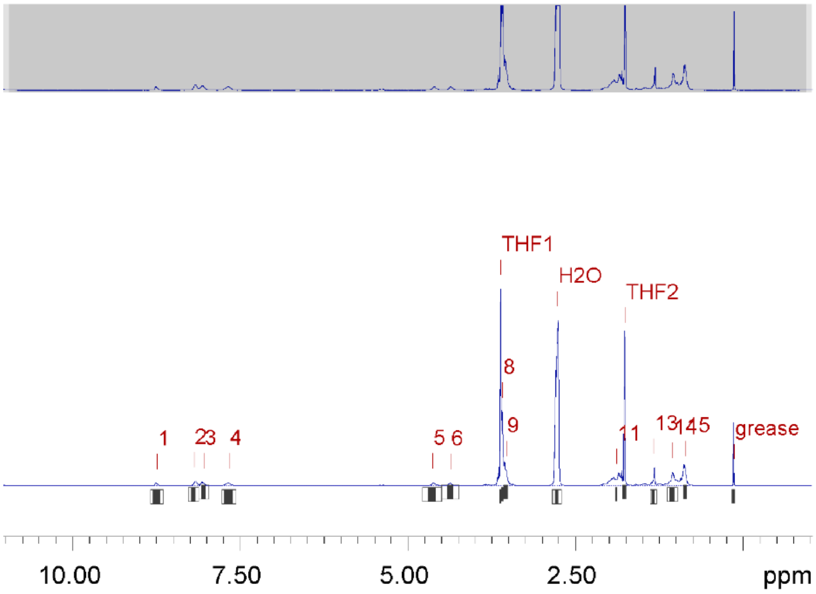
THF1
H2O
THF2
8
9
1
23
4
5 6
11
13
1415
grease
10.00
7.50
5.00
2.50
ppm

Dosy/Fit

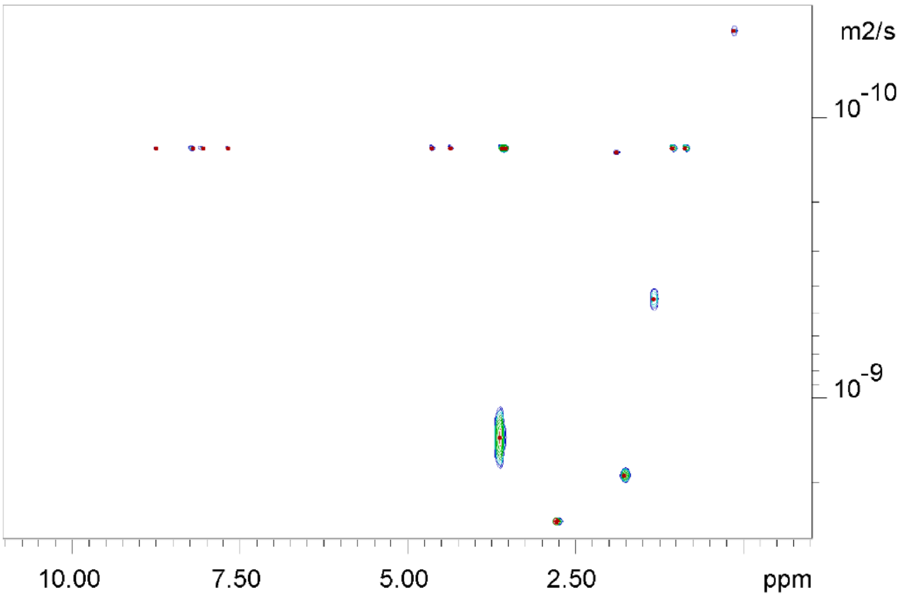
m2/s
10^{-10}
10^{-9}
10.00
7.50
5.00
2.50
ppm

Fitted function:	f (x) = Io * exp (-D * x^2 * gamma^2 * littleDelta^2 (bigDelta-littleDelta/3)* 10^4
used gamma:	26752 rad/(s*Gauss)
used little delta:	0.0036000 s
used big delta:	0.099900 s
used gradient strength:	variable
Random error estimation of data:	RMS per spectrum (or trace/plane)
Systematic error estimation of data:	worst case per peak scenario
Fit parameter Error estimation method:	from fit using arbitray y uncertainties
Confidence level:	95%
Used peaks:	peaks from //qut.edu.au/Documents/StaffHome/StaffGroupK$/kodurad/Documents/PHD/NMR/DOSY/DAK_042_C_6Grad C/4/pdata/1/peaklist1D.xml
Used integrals:	area integral
Used Gradient strength:	all values (including replicates) used

Peak name	F2 [ppm]	Io	error	D [m2/s]	error	fitInfo
grease	0.138	1.76e+09	1.732e+07	4.95e-11	1.652e-12	Done
THF1	3.616	1.60e+10	9.630e+08	1.40e-09	1.908e-10	Done
1	8.743	9.23e+08	6.117e+06	1.28e-10	2.024e-12	Done
THF2	1.763	1.82e+10	2.232e+08	1.91e-09	5.280e-11	Done
2	8.190	1.83e+09	1.346e+07	1.28e-10	2.245e-12	Done
H2O	2.770	5.92e+10	8.347e+07	2.72e-09	8.584e-12	Done
3	8.042	1.78e+09	7.758e+06	1.30e-10	1.343e-12	Done
4	7.668	1.76e+09	8.247e+06	1.30e-10	1.447e-12	Done
5	4.635	1.55e+09	1.763e+07	1.28e-10	3.450e-12	Done
6	4.358	1.50e+09	1.520e+07	1.30e-10	3.118e-12	Done
8	3.589	1.29e+10	6.519e+07	1.31e-10	1.567e-12	Done
9	3.536	7.17e+09	4.247e+07	1.29e-10	1.817e-12	Done
11	1.888	1.36e+09	1.071e+07	1.35e-10	2.512e-12	Done
13	1.336	2.81e+09	8.358e+07	4.49e-10	3.049e-11	Done
14	1.053	6.51e+09	4.015e+07	1.29e-10	1.887e-12	Done
15	0.860	5.63e+09	2.797e+07	1.31e-10	1.540e-12	Done

Current fit display

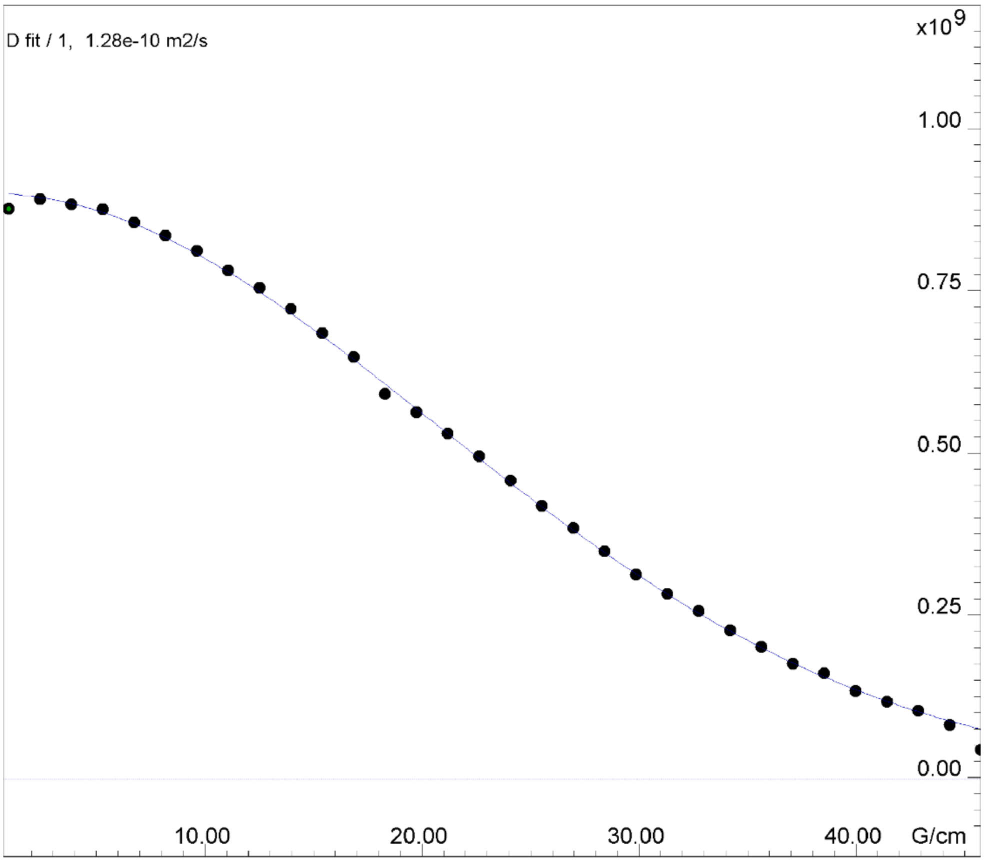

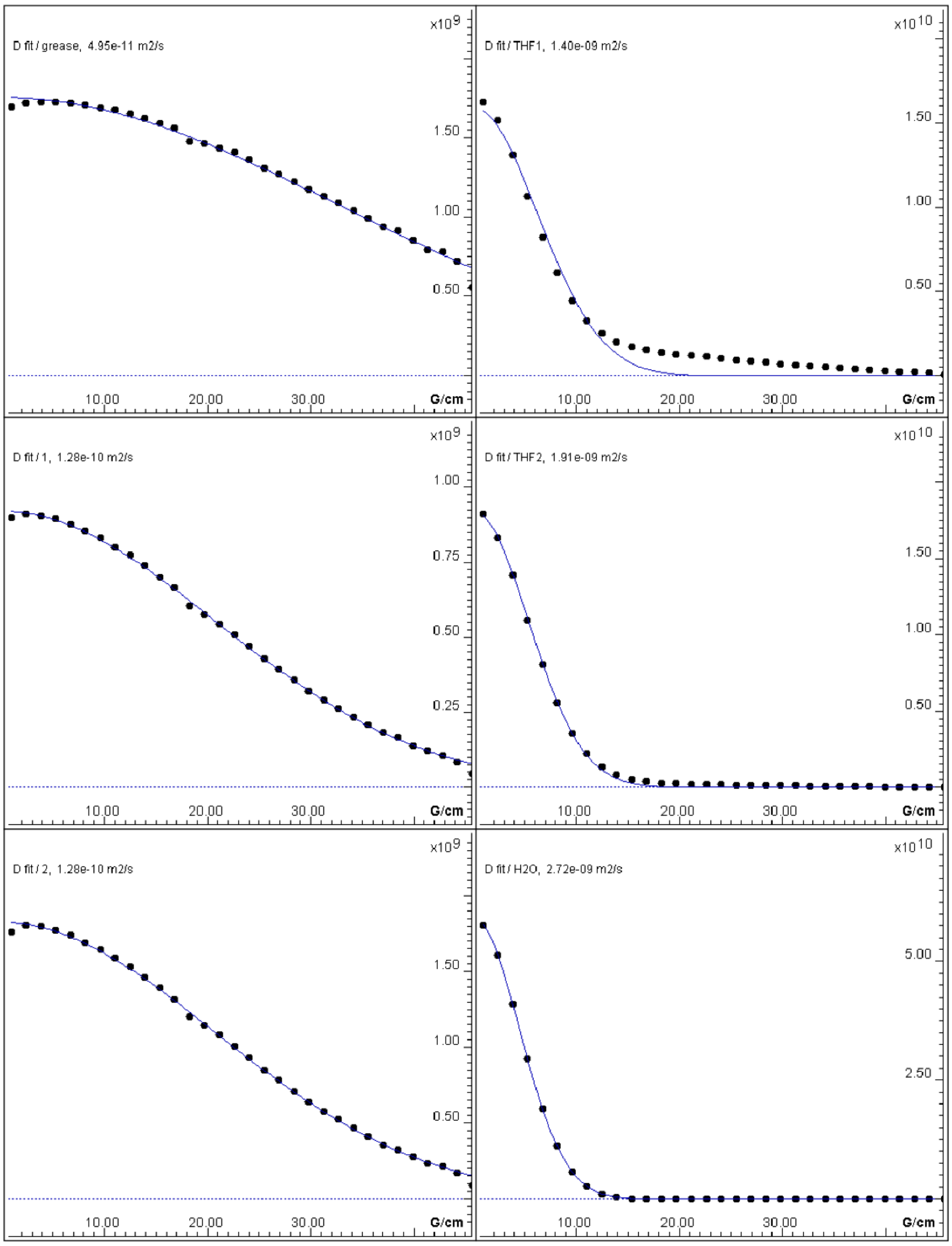
D fit / grease, 4.95e-11 m2/s
D fit / THF1, 1.40e-09 m2/s
D fit / 1, 1.28e-10 m2/s
D fit / THF2, 1.91e-09 m2/s
D fit / 2, 1.28e-10 m2/s
D fit / H2O, 2.72e-09 m2/s
G/cm

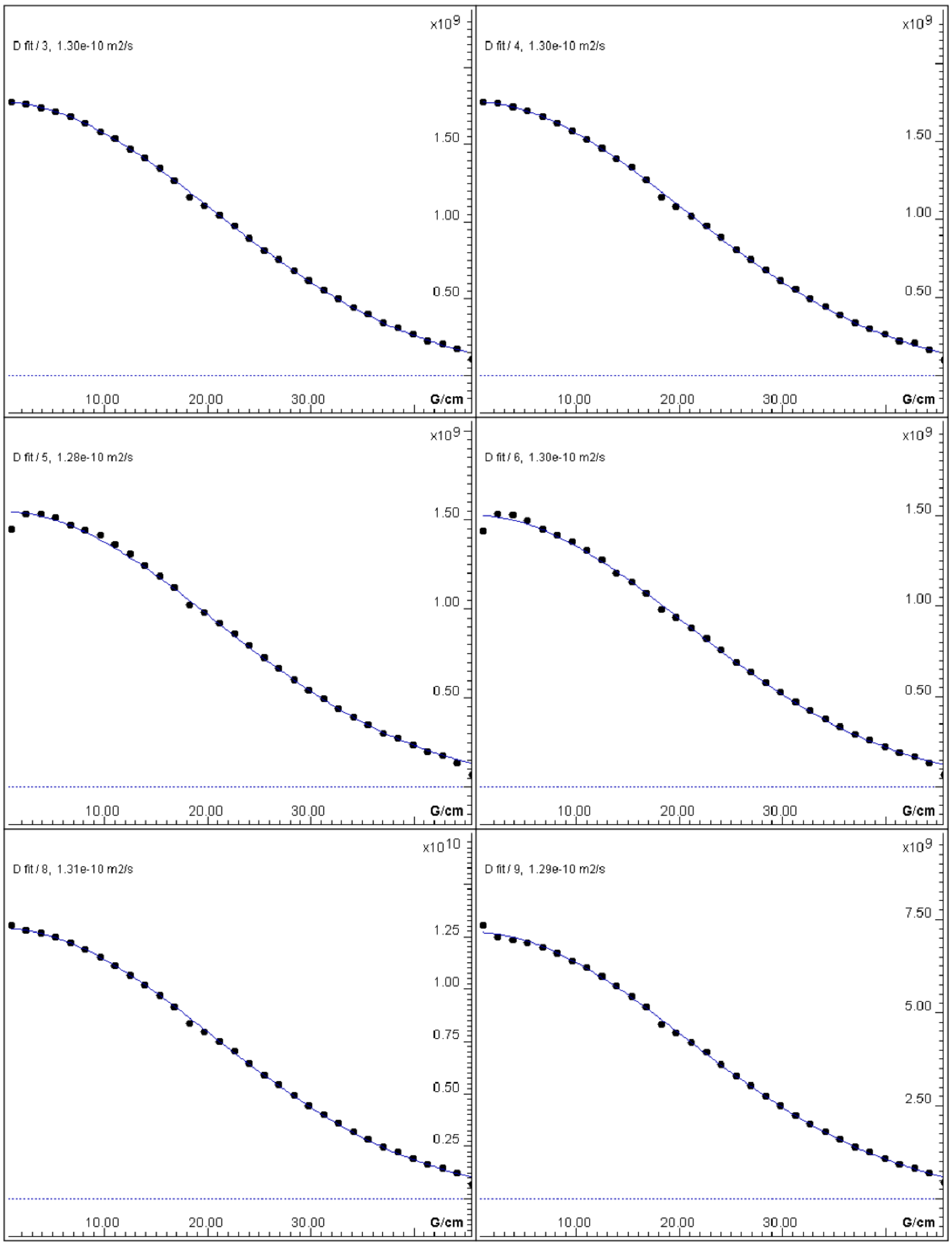
D fit / 3, 1.30e-10 m2/s
D fit / 4, 1.30e-10 m2/s
D fit / 5, 1.28e-10 m2/s
D fit / 6, 1.30e-10 m2/s
D fit / 8, 1.31e-10 m2/s
D fit / 9, 1.29e-10 m2/s
x10⁹
x10¹⁰
10.00
20.00
30.00
G/cm
0.25
0.50
0.75
1.00
1.25
1.50
2.50
5.00
7.50

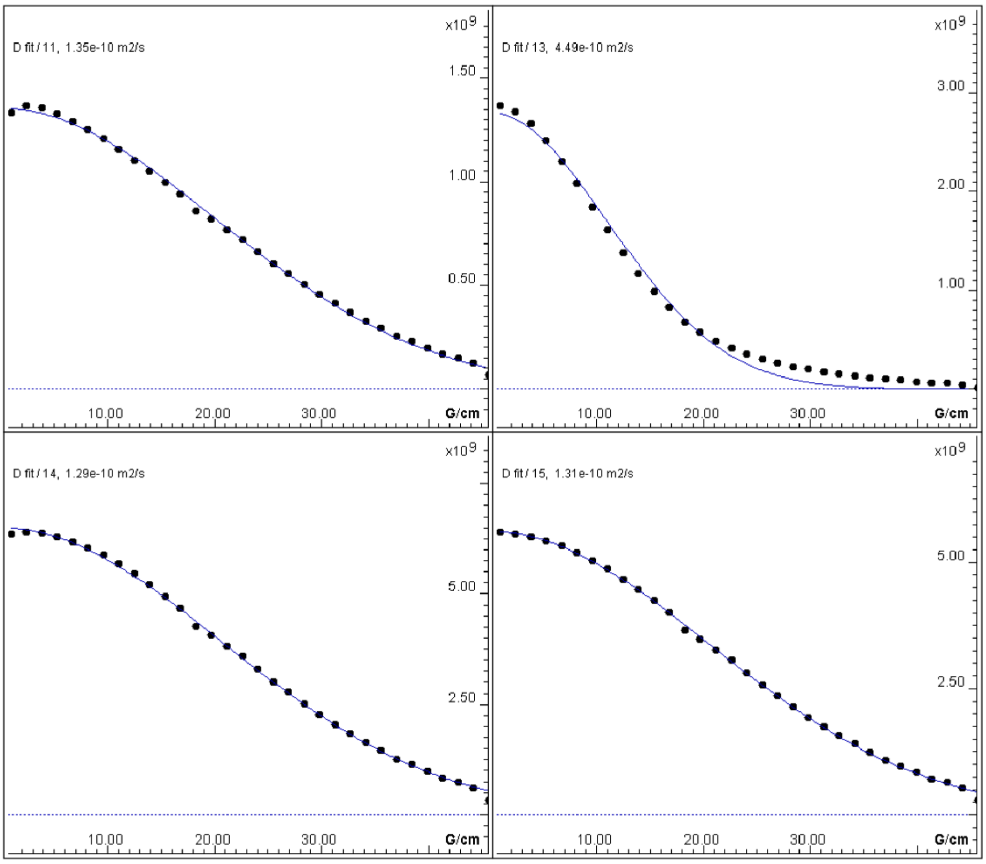
D fit / 11, 1.35e-10 m2/s
x10^9
1.50
1.00
0.50
10.00
20.00
30.00
G/cm
D fit / 13, 4.49e-10 m2/s
x10^9
3.00
2.00
1.00
10.00
20.00
30.00
G/cm
D fit / 14, 1.29e-10 m2/s
x10^9
5.00
2.50
10.00
20.00
30.00
G/cm
D fit / 15, 1.31e-10 m2/s
x10^9
5.00
2.50
10.00
20.00
30.00
G/cm

• Diffusion Analysis

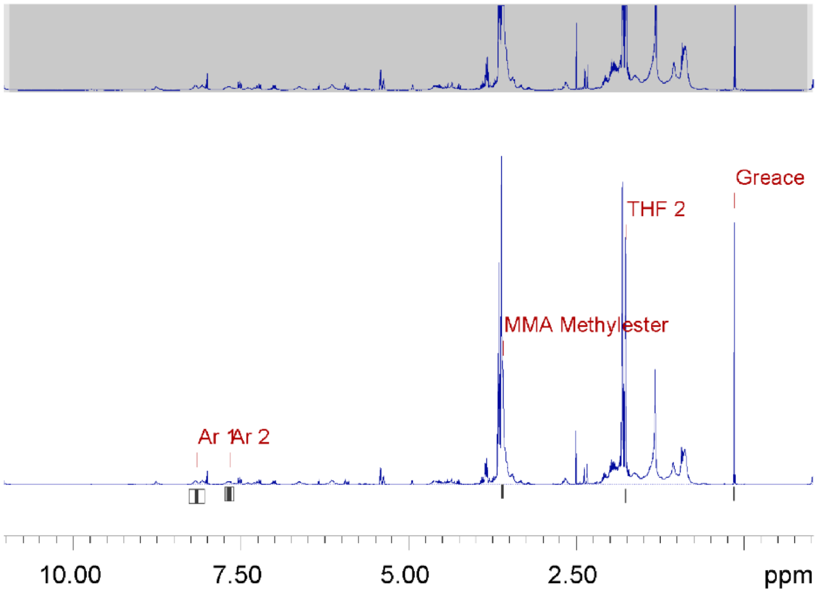

Dosy/Fit

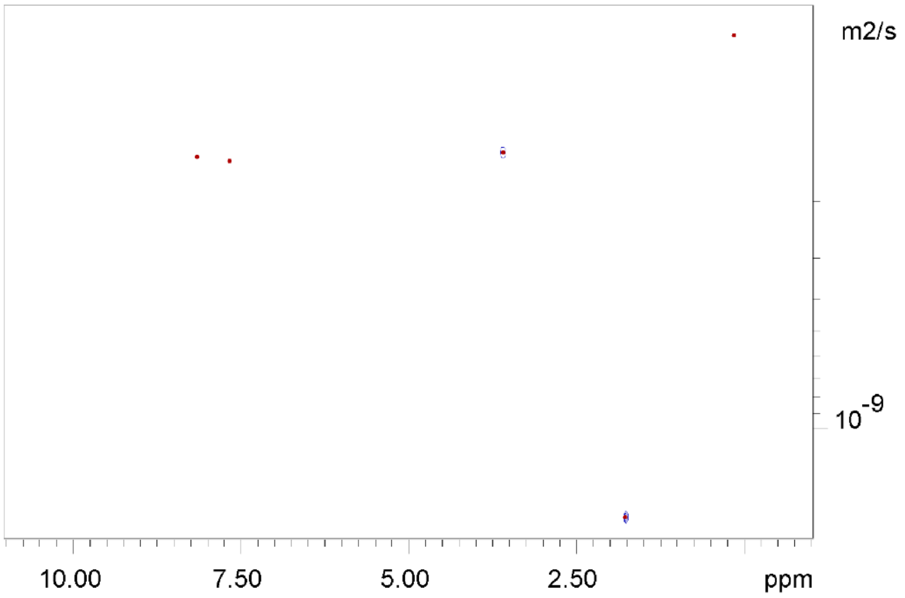

Fitted function:	f (x) = Io * exp (-D * x^2 * gamma^2 * littleDelta^2 (bigDelta-littleDelta/3)* 10^4
used gamma:	26752 rad/(s*Gauss)
used little delta:	0.0036000 s
used big delta:	0.099900 s
used gradient strength:	variable
Random error estimation of data:	RMS per spectrum (or trace/plane)
Systematic error estimation of data:	worst case per peak scenario
Fit parameter Error estimation method:	from fit using arbitray y uncertainties
Confidence level:	95%
Used peaks:	peaks from //qut.edu.au/Documents/StaffHome/StaffGroupK$/kodurad/Documents/PHD/NMR/DOSY/DAK_097/4/pdata/1/peaklist1D.xml
Used integrals:	area integral
Used Gradient strength:	all values (including replicates) used

Peak name	F2 [ppm]	D [m2/s]	error	fitInfo
MMA Methylester	3.591	1.43e-10	2.987e-12	Done
Greace	0.144	6.19e-11	1.452e-12	Done
Ar 1	8.152	1.44e-10	1.892e-12	Done
Ar 2	7.666	1.49e-10	2.324e-12	Done
THF 2	1.765	1.86e-09	4.366e-11	Done

Current fit display

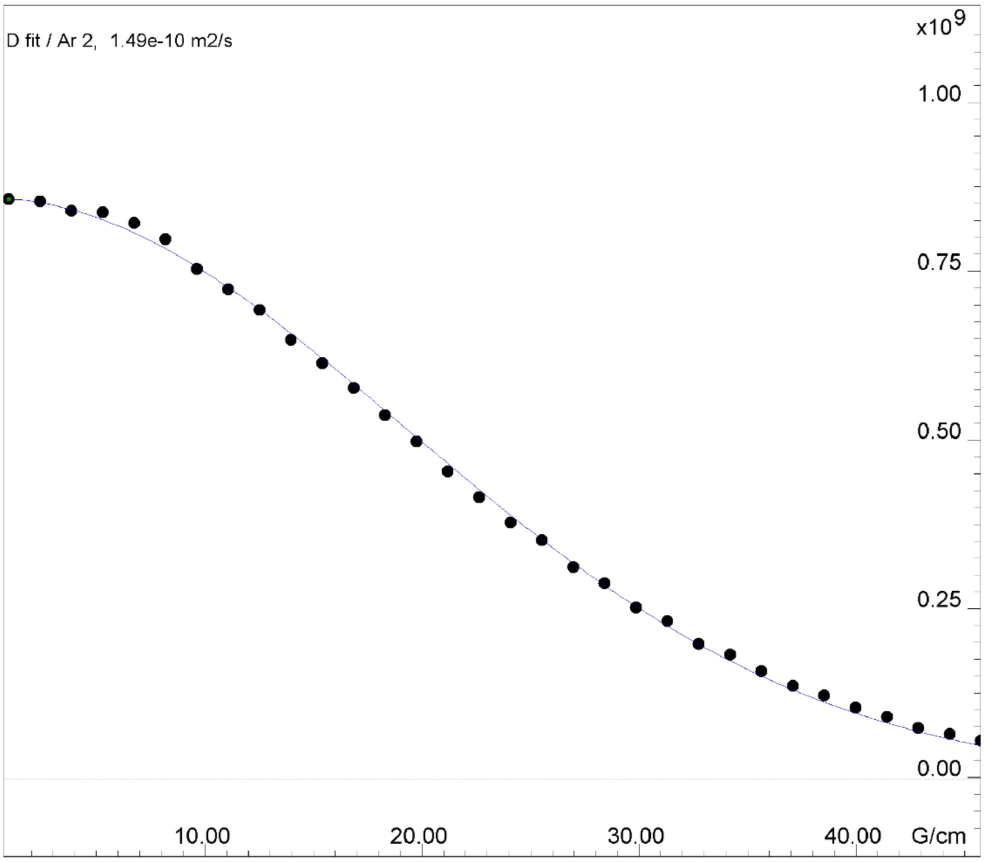

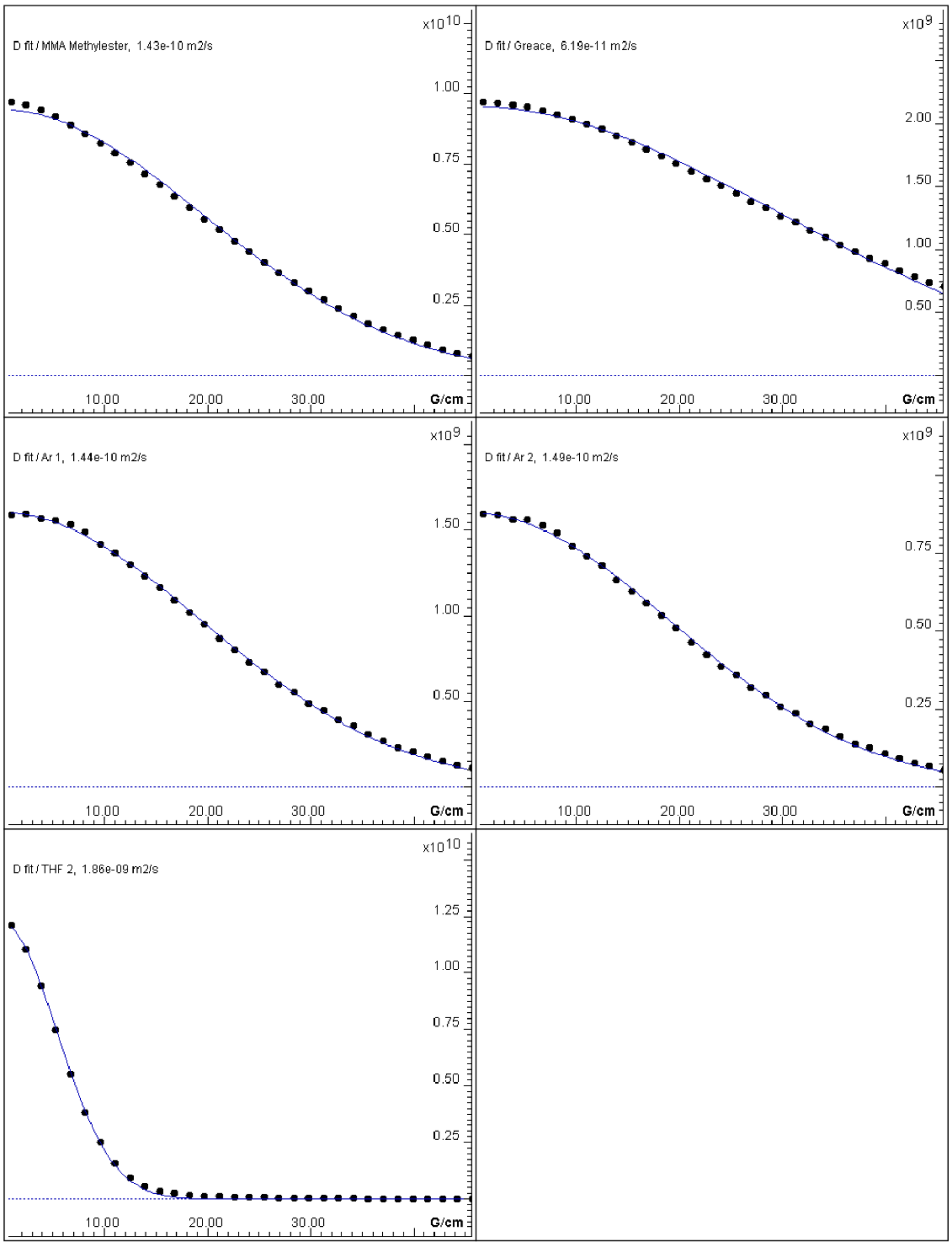
D fit / MMA Methylester, 1.43e-10 m2/s
x10^10
1.00
0.75
0.50
0.25
10.00
20.00
30.00
G/cm
D fit / Greace, 6.19e-11 m2/s
x10^9
2.00
1.50
1.00
0.50
D fit / Ar 1, 1.44e-10 m2/s
1.50
D fit / Ar 2, 1.49e-10 m2/s
D fit / THF 2, 1.86e-09 m2/s
1.25

• Diffusion Analysis

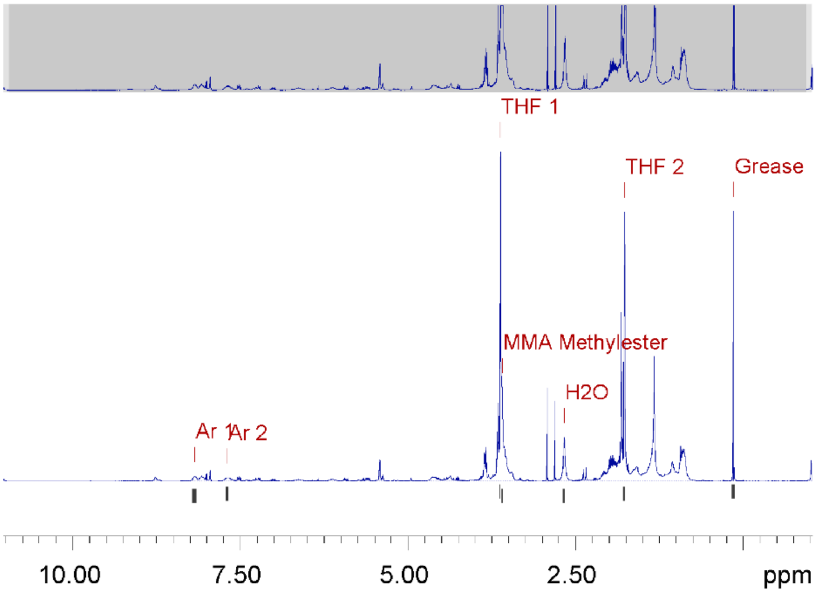

Dosy/Fit

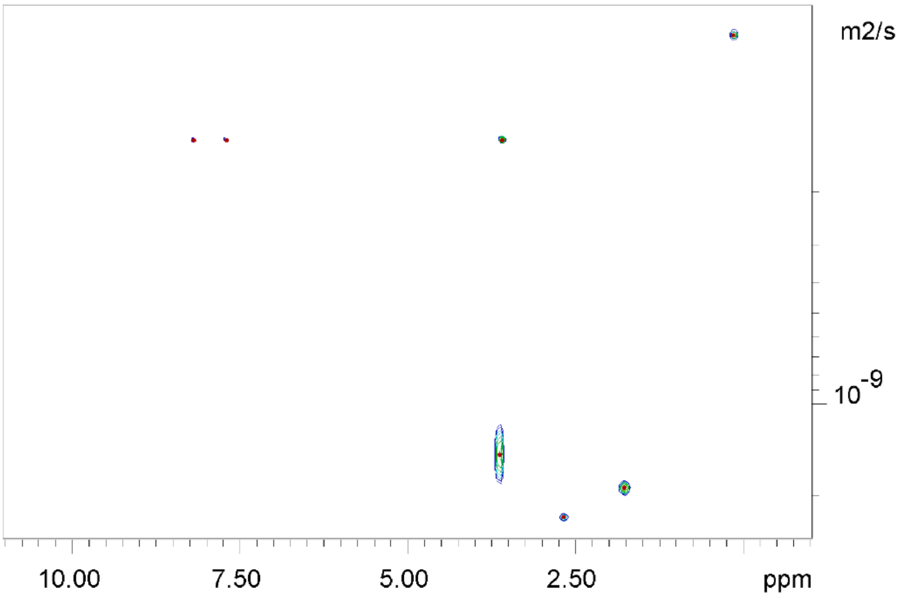

Friday, February 7, 2020 4:40:46 PM
kodurad@SEF-PA00144021
\\qut.edu.au\Documents\StaffHome\staffgroupK$\kodurad\Documents\PHD\NMR\DOSY\DAK_097_24h\final DAK_097_24h_4_1.pdf
Dynamics Center 2.6 (2019 Nov/26)

Fitted function:	f (x) = Io * exp (-D * x^2 * gamma^2 * littleDelta^2 (bigDelta-littleDelta/3)* 10^4
used gamma:	26752 rad/(s*Gauss)
used little delta:	0.0036000 s
used big delta:	0.099900 s
used gradient strength:	variable
Random error estimation of data:	RMS per spectrum (or trace/plane)
Systematic error estimation of data:	worst case per peak scenario
Fit parameter Error estimation method:	from fit using arbitray y uncertainties
Confidence level:	95%
Used peaks:	peaks from //qut.edu.au/Documents/StaffHome/StaffGroupK$/kodurad/Documents/PHD/NMR/DOSY/DAK_097_24h/4/pdata/1/peaklist1D.xml
Used integrals:	area integral
Used Gradient strength:	all values (including replicates) used

Peak name	F2 [ppm]	D [m2/s]	error	fitInfo
MMA Methylester	3.588	1.34e-10	1.314e-12	Done
Grease	0.145	6.05e-11	1.216e-12	Done
Ar 1	8.178	1.33e-10	1.468e-12	Done
THF 1	3.623	1.48e-09	2.060e-10	Done
H2O	2.668	2.37e-09	4.763e-11	Done
Ar 2	7.695	1.33e-10	2.025e-12	Done
THF 2	1.769	1.88e-09	4.047e-11	Done

Current fit display

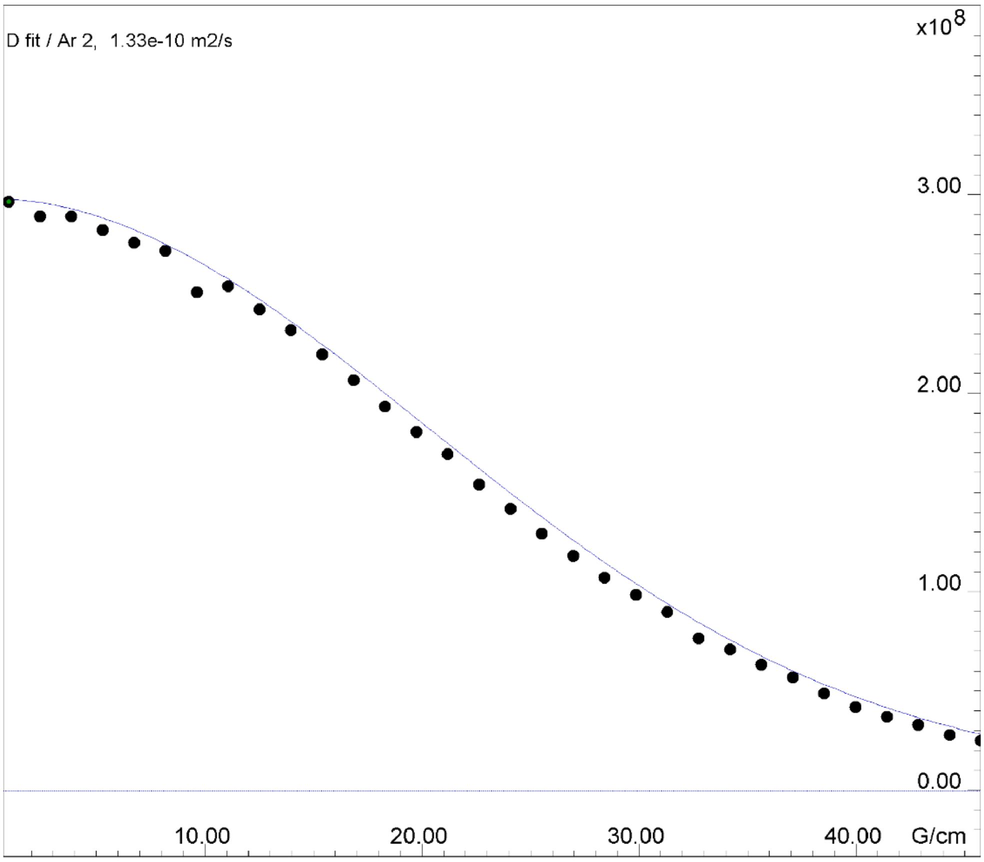

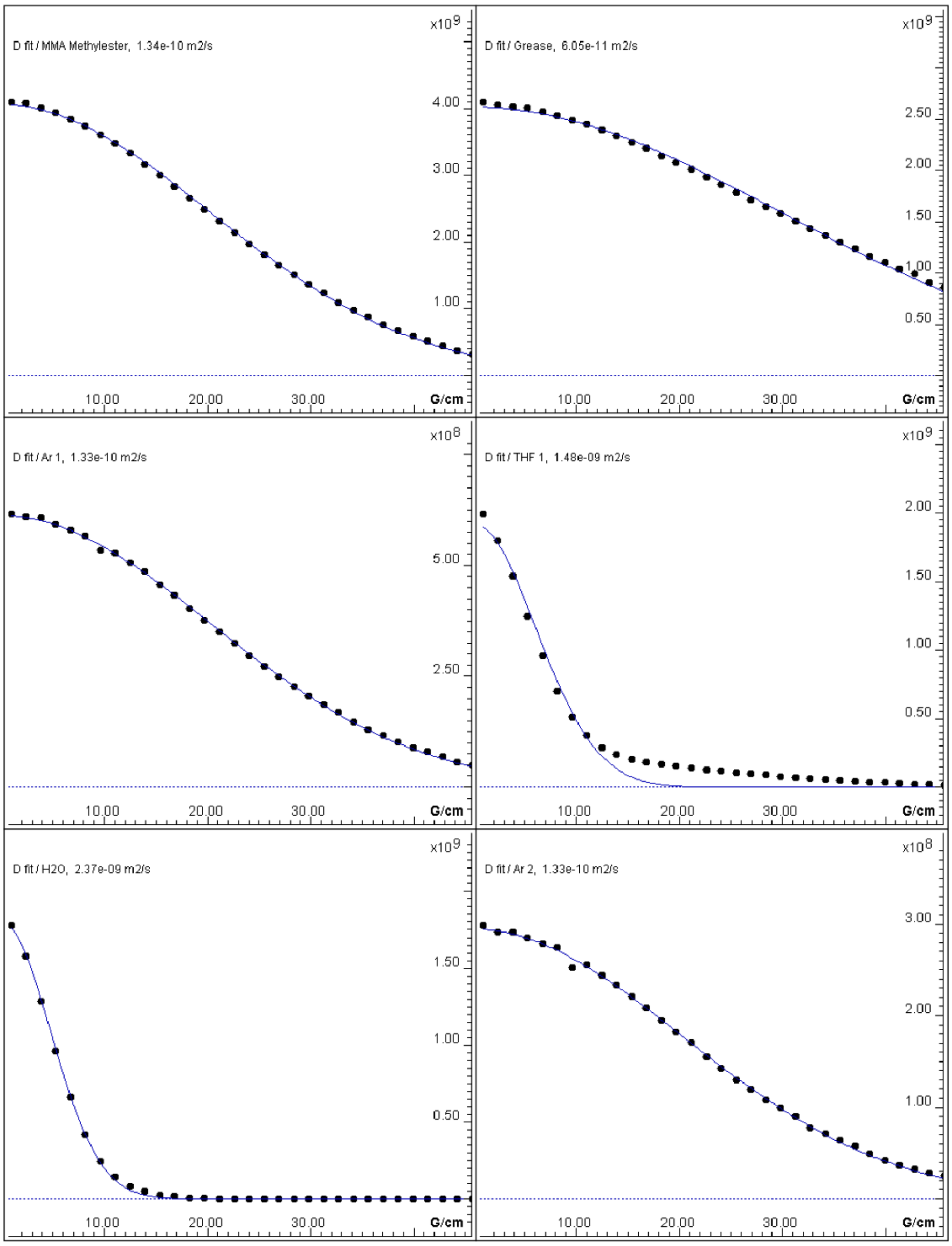
D fit / MMA Methylester, 1.34e-10 m2/s
x10^9
4.00
3.00
2.00
1.00
10.00
20.00
30.00
G/cm
D fit / Grease, 6.05e-11 m2/s
x10^9
2.50
2.00
1.50
1.00
0.50
10.00
20.00
30.00
G/cm
D fit / Ar 1, 1.33e-10 m2/s
x10^8
5.00
2.50
10.00
20.00
30.00
G/cm
D fit / THF 1, 1.48e-09 m2/s
x10^9
2.00
1.50
1.00
0.50
10.00
20.00
30.00
G/cm
D fit / H2O, 2.37e-09 m2/s
x10^9
1.50
1.00
0.50
10.00
20.00
30.00
G/cm
D fit / Ar 2, 1.33e-10 m2/s
x10^8
3.00
2.00
1.00
10.00
20.00
30.00
G/cm

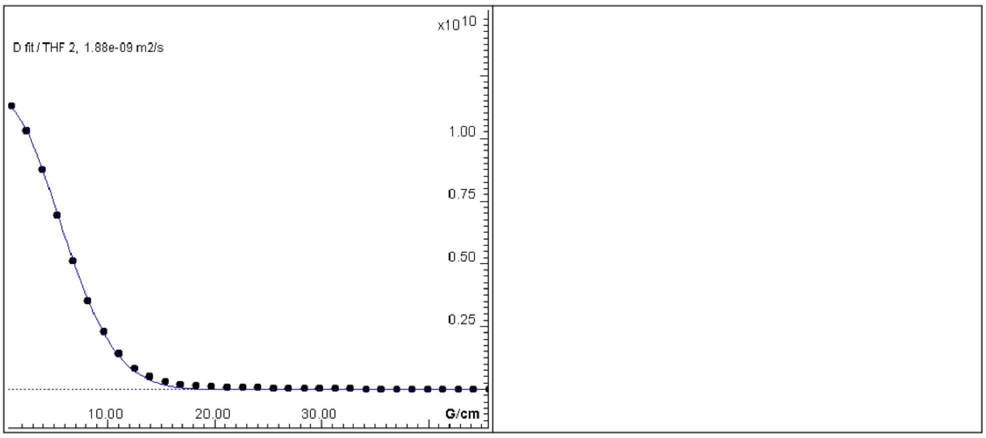
D fit / THF 2, 1.88e-09 m2/s
x10^10
1.00
0.75
0.50
0.25
10.00
20.00
30.00
G/cm
